# KLF5 inhibition potentiates anti-PD1 efficacy by enhancing CD8^+^ T-cell-dependent antitumor immunity

**DOI:** 10.7150/thno.82182

**Published:** 2023-02-21

**Authors:** Qi Wu, Zhou Liu, Zhijie Gao, Yao Luo, Fubing Li, ChuanYu Yang, Tiantian Wang, Xiangyu Meng, Haijun Chen, Juanjuan Li, Yanjie Kong, Chao Dong, Si Sun, Ceshi Chen

**Affiliations:** 1 Key Laboratory of Animal Models and Human Disease Mechanisms of the Chinese Academy of Sciences and Yunnan Province, KIZ-CUHK Joint Laboratory of Bioresources and Molecular Research in Common Diseases, Kunming Institute of Zoology, Chinese Academy of Sciences, Kunming, China; 2 Tongji University Cancer Center, Shanghai Tenth People's Hospital, Tongji University School of Medicine, Shanghai, China; 3 Department of Breast and Thyroid Surgery, Renmin Hospital of Wuhan University, Wuhan, Hubei, China; 4 Medical Faculty of Kunming University of Science and Technology, Kunming, China; 5 Academy of Biomedical Engineering, Kunming Medical University, Kunming 650500, China; 6 School of Life Science, University of Science & Technology of China, Hefei, 230027, Anhui, China; 7 Center for Single-Cell Omics and Tumor Liquid Biopsy, Zhongnan Hospital of Wuhan University, Wuhan, Hubei, China; 8 College of Chemistry, Fuzhou University, Fuzhou, Fujian 350108, China; 9 Department of Medical Oncology, The First Affiliated Hospital of Kunming Medical University, Kunming, China; 10 Department of Clinical Laboratory, Renmin Hospital of Wuhan University, Wuhan, Hubei, China; 11 The Third Affiliated Hospital, Kunming Medical University, Kunming 650118, China; 12 Pathology department, Shenzhen Second People's Hospital, the First Affiliated Hospital of Shenzhen University, Health Science Center, Shenzhen 518035, China

**Keywords:** KLF5, COX2, PD1 blocker, CD8^+^ T cell, breast cancer

## Abstract

**Background:** Immune checkpoint blockers (ICBs) are revolutionized therapeutic strategies for cancer, but most patients with solid neoplasms remain resistant to ICBs, partly because of the difficulty in reversing the highly immunosuppressive tumor microenvironment (TME). Exploring the strategies for tumor immunotherapy is highly dependent on the discovery of molecular mechanisms of tumor immune escape and potential therapeutic target. Krüppel-like Factor 5 (KLF5) is a cell-intrinsic oncogene to promote tumorigenesis. However, the cell-extrinsic effects of KLF5 on suppressing the immune response to cancer remain unclear.

**Methods:** We analyzed the immunosuppressive role of KLF5 in mice models transplanted with KLF5-deleted/overexpressing tumor cells. We performed RNA sequencing, immunohistochemistry, western blotting, real time-PCR, ELISA, luciferase assay, chromatin immunoprecipitation (ChIP), and flow cytometry to demonstrate the effects of KLF5 on CD8^+^ T cell infiltration and related molecular mechanism. Single-cell RNA sequencing and spatial transcriptomics analysis were applied to further decipher the association between KLF5 expression and infiltrating immune cells. The efficacy of KLF5/COX2 inhibitors combined with anti-programmed cell death protein 1 (anti-PD1) therapy were explored in pre-clinical models. Finally, a gene-expression signature depending on KLF5/COX2 axis and associated immune markers was created to predict patient survival.

**Results:** KLF5 inactivation decelerated basal-like breast tumor growth in a CD8^+^ T-cell-dependent manner. Transcriptomic profiling revealed that KLF5 loss in tumors increases the number and activated function of T lymphocytes. Mechanistically, KLF5 binds to the promoter of the COX2 gene and promotes COX2 transcription; subsequently, KLF5 deficiency decreases prostaglandin E2 (PGE2) release from tumor cells by reducing COX2 expression. Inhibition of the KLF5/COX2 axis increases the number and functionality of intratumoral antitumor T cells to synergize the antitumorigenic effects of anti-PD1 therapy. Analysis of patient datasets at single-cell and spatial resolution shows that low expression of KLF5 is associated with an immune-supportive TME. Finally, we generate a KLF5/COX2-associated immune score (KC-IS) to predict patient survival.

**Conclusions:** Our results identified a novel mechanism responsible for KLF5-mediated immunosuppression in TME, and targeting the KLF5/COX2/PGE2 axis is a critical immunotherapy sensitizer.

## Background

Recently, cancer immunotherapy has achieved remarkable breakthroughs in clinical practice. The clinically developed immunotherapeutic strategies comprise inhibitory immune checkpoint blockers (ICBs), enhanced costimulators, oncolytic viruses, various vaccines and adoptive cell therapies 1. Programmed cell death protein 1/programmed cell death ligand 1 (PD1/PD-L1) is considered a main immune checkpoint, and its blockers have been approved by the US Food and Drug Administration (FDA) 2. In recent years, atezolizumab (PD-L1 inhibitor) combined with paclitaxel chemotherapy has achieved efficacy in PD-L1-positive triple-negative breast cancer (TNBC) patients 3. Likewise, neoadjuvant toripalimab with or without celecoxib resulted in a favorable pathological complete response rate in patients with mismatch repair-deficient or microsatellite instability-high, locally advanced, colorectal cancer 4. However, PD1/PD-L1 immune checkpoint blockade only benefits a small subset of patients and fails to generate durable responses in most patients 5, 6. Intrinsically and extrinsically immunosuppressive mechanisms endow tumors with the capacity to resist anticancer therapies 7, 8. Hence, developing comprehensive strategies or fire-new drugs is crucial to form an immune-supportive microenvironment and surmount resistance to immunotherapy.

Krüppel-like Factor 5 (KLF5), a member of the Krüppel-like factor family, controls essential cellular processes, including proliferation, differentiation, and migration 9. Structurally, KLF5 has a triple zinc-finger DNA-binding domain at its C-terminus, which mainly binds to CACC or GC boxes in DNA and further modulates the transcription of downstream target genes, such as *fibroblast growth factor-binding protein 1 (FGF-BP1)* 10*, p27* 11*, Cyclin D1* 12*, TNFAIP2* 13*, mPGES1* 14*, Slug* 15* and IGFL1* 16. KLF5 is an oncogene in basal-like breast cancer (BLBC), colorectal cancer and pancreatic cancer relevant to tumor stemness, proliferation, invasion, metastasis, and the tumor microenvironment (TME) 9, 17, 18. Furthermore, KLF5 is a potent therapeutic target for BLBC and other cancers. Our previous studies have shown that metformin, mifepristone, the bromodomain 4 (BRD4) inhibitors 19, mithramycin A 20, CDK7 inhibitor 19, PRMT5 inhibitor 21, RSK2 inhibitor 22, and HDAC inhibitor 23 retard tumor growth by downregulating KLF5 expression 10, 19, 24. In advanced colorectal cancer, mesenchymal stromal cell-derived CCL7 stimulated the acetylation of KLF5 by p300, subsequently acetylated KLF5 and transcriptionally activated CXCL5 expression to facilitate tumor metastasis 25. Likewise, lysine demethylase 3A (KDM3A) could upregulate the transcription of epidermal growth factor receptor (EGFR) by recruiting KLF5 and SMAD family member 4 (SMAD4). KLF5 knockdown sensitized tumors to PD-1 blockade by increasing CD4^+^ and CD8^+^ T cells and reducing myeloid-derived suppressor cells (MDSCs) 26. Given the functions of KLF5 in the tumor-immune microenvironment (TIME), knowing the mechanisms by which KLF5 influences the composition of the TIME is crucial.

Cyclooxygenases (COXs), as catabolic enzymes, enable the conversion of arachidonic acids to prostaglandin G2 (PGG2) and H2 (PGH2), which are further transformed to prostaglandin I2 (PGI2), prostaglandin D2 (PGD2), and prostaglandin E2 (PGE2). COXs primarily comprise constitutive COX1 and inducible COX2^27^. PGE2 plays a pivotal role in various human diseases, such as cardiovascular disease, cancer, and neurological diseases 28-30. In the tumor microenvironment, PGE2 can be released by multiple cell types, such as tumor cells, cancer-associated fibroblasts and MDSCs 28. PGE2 production is regulated by diverse inflammatory stimuli and transcription factors, including KLF5 and p65^14^, ^28^. Specifically, KLF5 binds to the *mPGES1* gene proximal promoter and activates its transcription to promote PGE2 synthesis 14. As a proinflammatory lipid metabolite, PGE2 interacts with a family of G protein-coupled receptors—E-type prostaglandin receptors 1-4 (EP1-4) 31. Notably, PGE2 exerts a protumorigenic effect by stimulating the proliferation and metastasis of neoplastic cells and tumor angiogenesis 28. Likewise, PGE2 has a pivotal immunosuppressive effect via multiple mechanisms, including directly impairing the proliferation and activation of NK cells and effector T cells, suppressing the antigen presentation of dendritic cells and increasing the infiltration of MDSCs and regulatory T cells (Tregs) 31. Additionally, inhibition of the COX2/mPGES1/PGE2 axis or EPs antagonists enhances the efficacy of PD-1 blockers to improve antitumor activity in various tumor models 32-34. Therefore, specific interruption of PGE2 generation or antagonism of its receptors may be used as adjuvants to synergize with immune-targeting drugs.

In this study, we found that KLF5 deficiency inhibits progressive tumor growth and enhances antitumor immunity in a CD8^+^ T-cell-dependent manner. Mechanistically, KLF5 promotes PGE2 release by transcriptionally activating COX2 expression. Additionally, KLF5 knockdown, a KLF5 inhibitor or a COX2 selective inhibitor, synergized with the efficacy of the anti-PD1 blocker by increasing the infiltration and activating function of CD8+ T cells. Ultimately, we identified a gene signature that integrates the KLF5/COX2 axis and proliferation and activity of CD8^+^ T cells. This gene signature score showed independent prognostic value in BLBC.

## Materials and methods

### Cell culture and reagents

The mouse cancer cell lines TC1, MCA205, MC38, CT26, EMT6 and 67NR were obtained from Guido Kroemer's lab. The abovementioned mouse cancer cells, mouse breast cancer lines 4T1 and E0771 and human breast cancer MDA-MB-231 cell lines were maintained in Dulbecco's modified Eagle's medium (DMEM) supplemented with 10% (v/v) fetal bovine serum (FBS) at 37 °C in a humidified atmosphere with 5% CO_2_. Human breast cancer HCC1806 cells were cultured in Roswell Park Memorial Institute (RMPI)-1640 medium supplemented with 10% FBS. Lipopolysaccharide (LPS) and celecoxib (CEL) were purchased from MedChemExpress (Shanghai, China). FZU-00,004 was synthesized by Haijun Chen (College of Chemistry, Fuzhou University, China).

### Lentivirus preparation and transfection

KLF5 siRNA and cDNA lentivirus were obtained from GeneChem Biotechnology (Shanghai, China). Cells were cultured at 5 × 10^5^ cells/well in 6-well plates. After incubation for 24 h, the cells were transfected with the aforementioned lentivirus and control vectors (GeneChem Biotechnology, China) following the manufacturer's instructions. Selection was performed using puromycin (1 μg/mL; Sigma) in cell culture media for 48 h after transfection. Cell lysates were then collected, and protein expression was detected by Western blotting (WB). The sequence information is provided in Table S1.

### Patients

A total of 67 formalin-fixed paraffin-embedded (FFPE) colon cancer tissue samples were obtained from Renmin Hospital of Wuhan University. All the patients involved in the study provided written informed consent. Patients did not receive financial compensation. Clinical information was extracted from medical records and pathology reports, and the detailed clinicopathological characteristics of the patients are shown in Table S4. The patients were all followed-up for at least 38.1 months from the date of the first diagnosis. All the procedures were performed in accordance with the Declaration of Helsinki and relevant guidelines and local regulations. The study was approved by the Institutional Ethics Committee of Renmin Hospital of Wuhan University (approval no. 2018K-C09).

### ELISA

Tissue samples (~30 mg) were dissociated in tubes containing 1 mL of radio immunoprecipitation assay buffer (RIPA) lysis buffer using a homogenizer (Servicebio, China) at 6,500 rpm for 5 min, followed by centrifugation at 14,000 × g for 15 min to collect the supernatant containing soluble proteins. For cells, the media were collected via centrifugation at 14,000 × g for 15 min at 4 ℃. The PGE2 level was measured using a mouse PGE2 ELISA kit (CSB-E07966m; CUSABIO) following the manufacturer's instructions. The PGE2 levels were standardized by the tissue weight or the cellular protein concentration.

### Western blotting

The protein extracts were dissolved in RIPA buffer for 30 min on ice, and then the samples were centrifuged at 12,000 × g for 15 min to collect the supernatant containing soluble proteins. The protein concentration was measured using the BCA Assay (Bio-Rad, Hercules, CA, USA). The protein solution was mixed with 4×loading buffer and heated at 100 °C for 10 min before being subjected to WB. The total protein samples (~ 30 μg) were subjected to SDS‒PAGE and then blotted onto 0.2 μM polyvinylidene fluoride (PVDF) membranes (#1620177; Bio-Rad). The membranes were blocked with 0.05% Tween 20 (#P9416; Sigma Aldrich) v:v in Tris-buffered saline (TBS) (TBST) (#ET220; Euromedex) supplemented with 5% nonfat powdered milk (w:v in TBS), followed by overnight incubation at 4 °C with primary antibodies specific for KLF5 (#AF3758; 1:1000; R&D Systems), COX2 (#66351-1-Ig; 1:1000; Proteintech), CyclinD1 (#55506; 1:1000; Cell Signaling Technology) and Vinculin (#13901; 1:2000; Cell Signaling Technology). The membranes were washed with TBST three times for 10 min before incubation with HRP-conjugated secondary antibody for 1 h at room temperature. Next, the membranes were washed again and subjected to chemiluminescence detection using the Amersham ECL Prime detection reagent kit (GE Healthcare, Piscataway, NJ, USA) on an ImageQuant LAS 4000 software-assisted imager.

### RNA extraction and quantitative RT‒PCR

Total mRNA was collected by TRIzol reagent (Invitrogen). Reverse transcription was performed using the TaqMan® mRNA Reverse Transcription Kit (Vazyme, China), and mRNA levels were quantified using RT Real-Time SYBR Green/Rox PCR master mix (Vazyme, China) on the ABI-7900 system. The mRNA primer sequences are provided in Table S2.

### Chromatin immunoprecipitation assay

ChIP was performed using 67NR wt/KLF5-3F OV and HCC1806 cells following a protocol provided by Abcam (Cambridge, MA, USA). The diluted DNA-protein complex (25 μg protein) was incubated with different antibodies (anti-KLF5 Ab and goat IgG) overnight at 4 ℃ in the presence of herring sperm DNA and protein A/G beads or anti-Flag magnetic beads. PCR was performed on 67NR using primers for the PTGS2 promoter to amplify the -929 to -918 region: 5'-CAAGAACGTACAGTTTAGTTG-3' (forward) and 5'-TTGCCTAGAGAGGTGATGTTTTTGAT-3' (backward); a nonspecific KLF5-binding site: 5'-GGCAGCTTATAACTTTCTATAACTATAGT-3' (forward) and 5'-TATTTATTTATTTATTTATTTATTTATTTATTTTGTGTG-3' (backward). For HCC1806, the primer sequences were as follows: the putative KLF5-binding site, 5'-CATAAAACATGTCAGCCTTTCTTAACCTTAC-3' (forward) and 5'-AATCTGAGCGGCCCTGAGGTC-3' (backward); a nonspecific KLF5-binding site: 5'-AGTTCTTTGATTAAGGTAGTAGTTACAC-3' (forward) and 5'-AACCAGGAAACTGATCTTGGTA-3' (backward).

### Dual luciferase assay

The COX2 proximal promoters were amplified using normal human DNA and mouse genomic DNA as templates. The PCR products were cloned into pGL3-BASIC (Promega, Madison, WI, USA). The inserts were confirmed by DNA sequencing. 293T cells were seeded into 24-well plates at 1×10^5^ cells per well. The next day, the cells were transfected in triplicate with the *COX2* gene promoter reporter constructs (500 μg per well) and an internal control pRL-TK (50 μg per well). Twenty-four hours after transfection, the cells were infected with a GFP control adenovirus and a KLF5 adenovirus for 4 h (~50% cells were infected under a fluorescence microscope). At 24 h after infection, luciferase activities were measured using the dual luciferase reporter assay system (Promega).

### Immunohistochemistry

A cohort of 67 human colon cancer specimens was collected from Renmin Hospital of Wuhan University from 2016 to 2017. Immunohistochemistry (IHC) staining was performed, and the staining results were scored using ImageJ software as previously described^35^. The infiltrating level of CD8^+^ cells was counted per square millimeter in each colon cancer specimen, while the protein expression level of KLF5 was described by the percentage of positive cells calculated using ImageJ software. The optimal cutoff values for all expression levels were determined using X-tile Software.

### Mouse models

All experiments involving animals were handled according to the protocol (SMKX-20160305-08) approved by the Animal Ethics Committee of the Kunming Institute of Zoology, CAS. All the mice were maintained in a temperature-controlled and pathogen-free environment with 12 h light/dark cycles and access to food and water ad libitum. All the animal experiments were performed in accordance with relevant guidelines and local regulations.

For virus-induced tumorigenesis, FVB/N mice carrying Klf5 alleles flanked by LoxP sites (Klf5^fl/fl^) have been described previously 15. The lentivirus carrying polyoma middle T-antigen (PyMT) or PyMT-Cre was intraductally injected into different sides of the same FVB/N Klf5^fl/fl^ mice at 5 weeks of age. After being isolated and dissociated, the tumors were further cultivated in DMEM/Ham's F-12 (50/50) medium containing 10% FBS. After verification of the Klf5 levels, the neoplastic cells were inoculated into the mammary fat pads of FVB/N mice. For tumor growth experiments, six-week-old female BALB/c mice were purchased from SJA Laboratory Animal Co., Ltd. (Hunan, China). Mouse mammary carcinoma EMT6 wild-type cells (3 × 10^5^) or EMT6 Klf5-knockdown cells (3 × 10^5^), 67NR wild-type cells (4 × 10^6^) or Klf5-overexpressing cells (4 × 10^6^), mouse colon cancer CT26 cells (5 × 10^5^) or CT26 Klf5-knockdown cells (5 × 10^5^) were subcutaneously injected into BALB/c hosts. When tumors grew to approximately 20 mm^3^ in volume, the mice were treated with CEL dissolved in corn oil (30 mg/kg, gavage daily for two weeks), FZU-00,004 (dissolved in 5% DMSO, 40% PEG300, 5% Tween 80, and 45% PBS; 1 mg intraperitoneal injection) or an equivalent volume of vehicle alone or in combination with 200 μg of anti-Pd-1 antibody (Clone 29 F.1A12; BioXcell, West Lebanon, NH, USA). The mouse weight and tumor growth were monitored and documented on subsequent days. The tumor area was defined as (longest diameter) × (shortest diameter) × 4/π and was measured once every 3 days using a Vernier caliper. Animals were sacrificed when the tumor size reached the endpoint or signs of obvious discomfort were observed following the advice of the Ethical Committee.

### Ex vivo phenotyping of the tumor immune infiltrate

The tumors were harvested, weighed and transferred on ice into gentle tubes containing 1 mL of RPMI medium. The tumors were dissociated first mechanically with scissors and then enzymatically using DNase I/Collagenase IV with shaking (> 200 rpm) at 37 ℃ for 1 h. The dissociated bulk tumor cell suspension was resuspended in RPMI 1640, sequentially passed through a 70 μm Smart-Strainer and washed twice with PBS. Finally, bulk tumor cells were resuspended in PBS at a concentration corresponding to 250 mg of the initial tumor weight per ml. Intracellular cytokine samples were restimulated with 200 μl of stimulation medium with brefeldin A (#423303; BioLegend) ex vivo for 5 h. Cell viability was determined using the LIVE/DEAD® Fixable UV Dead Cell dye (Thermo Fisher Scientific) to discriminate viable cells from damaged cells. Before staining tumor-infiltrating lymphocytes (TILs) for flow cytometry analysis, the samples (~50 mg) were incubated with anti-mouse Cd16/Cd32 (clone 2.4G2; Mouse BD Fc Block; BD Pharmingen) to block the Fc receptors. Surface staining of murine immune cell populations infiltrating the tumor was performed using the following fluorochrome-conjugated antibodies: anti-Cd45-BV650, anti-Cd3-Percp-cy5.5, anti-Cd8-FITC, anti-Cd4-PE, anti-Cd25-APC/Cy7, anti-Cxcr6-PE/Cy7 and anti-Pd-1-BV510 (BioLegend). Next, the cells were fixed and permeabilized in Foxp3 Fix/Perm buffer (BioLegend) and stained for intracellular Foxp3 (anti-Foxp3-BV421) and Ifnγ (anti-Ifnγ-APC). Finally, stained samples were run through a flow cytometer (LSR Fortessa; BD). The data were acquired using BD FACS-Diva software (BD Biosciences) and analyzed using FlowJo software (TreeStar). Absolute counts of leukocytes and tumor cells were normalized considering the following parameters: weight of the harvested tumor and total volume of the dissociated tumor cell suspension (cell concentration typically set to 250 mg/mL in PBS), proportion of the whole cell suspension and proportion of the cell suspension used for cytometry 36.

### Single-cell mRNA sequencing and analysis

Single-cell RNA-seq data were obtained from our previous data (GSE198745) and the public dataset (GSE176078) in Gene Expression Omnibus (GEO). Downstream single-cell data analyses were conducted using the Seurat package in R. Each sample was individually quality checked. Cells were filtered using the following criteria: at least 200 detected genes and no more than 15% mitochondrial reads per cell. Cells with extremely high numbers of reads or genes detected were filtered to minimize the occurrence of doublets. Genes expressed in fewer than 3 cells for individual samples were filtered. Multiple single-cell sample integration and batch effect correction were performed using the mutual nearest neighbors (MNN) method and “RunFastMNN” function from the SeuratWrappers package. The principal component dimensions 1:15 were used for all dimension reduction and integration steps. We conducted principal component analysis (PCA) on the normalized expression matrix using the top 2000 highly variable genes identified by the ''FindVariableGenes'' function in Seurat. For dimensionality reduction visualizations, we used the uniform manifold approximation and projection (UMAP) algorithm. Finally, the clusters were compared pairwise using the “FindAllMarkers” function to detect the cluster-specific expressed genes, which were used to achieve annotations for the clusters. We chose 6 triple-negative breast cancer (TNBC) patients (CID4523, CID4515, and CID4465 in the KLF5-high group and CID44041, CID4495, and CID4513 in the KLF5-low group) using paired bulk RNA-seq for subsequent immune cell analysis. The identification of diverse T-cell subpopulations referred to a single-cell resolved pancancer study of tumor-infiltrating T cells by Zhang et al. 37. The gene signatures of 186 metabolic and signaling pathways were curated from the KEGG subset of canonical pathways from the C2 collection using MSigDB. Single-cell signature scores were calculated using the Gene Set Variation Analysis (GSVA) method and GSVA package from Bioconductor. The differential metabolic and signaling pathways in the KLF5-high and KLF5-low groups were computed using the limma package.

### Spatial transcriptomics

The spatially resolved transcriptomic data and images of breast cancer patients in a previous study are available in GEO (GSE198745). Additionally, the public spatially resolved transcriptomics data and images of 4 TNBC patients (CID4465, CID44971, 1142243F, 1160920F) could be obtained from the Zenodo data repository (https://doi.org/10.5281/zenodo.4739739). The basal signature score was computed using GSVA based on the basal cell signature genes (KRT5, KRT14, KRT17). The CD4^+^ and CD8^+^ T signature scores were computed using GSVA based on the CD4^+^ and CD8^+^ T-cell signature genes (CD3D and CD4 for CD4^+^ T cells, CD3D and CD8A for CD8^+^ T cells).

### Bioinformatic Analysis of Patient Datasets

A total of 360 TNBC patients with RNA-seq data were obtained from Fudan University Shanghai Cancer Center (FUSCC) (https://www.biosino.org/node/analysis/detail/OEZ000398). The data of breast cancer patients in The Cancer Genome Atlas (TCGA) were downloaded from UCSC Xena (http://xena.ucsc.edu/), and TNBC patients were selected using the PAM50 classifier. For the RNA-seq data of FUSCC, after transforming the transcriptomics data to normalized transcripts per million values (TPM), we performed differential analysis of TGFB1-low patients grouped by the expression level of KLF5 by the limma package. Gene Ontology (GO), Kyoto Encyclopedia of Genes and Genomes (KEGG), and Gene Set Enrichment Analysis (GSEA) pathway analyses were executed using the clusterProfiler package. The abundance of diverse immune subpopulation infiltration was estimated using TIMER2.0 (http://timer.comp-genomics.org/). The 'cancer-promoting (CP)' and 'cancer-inhibitory (CI)' inflammatory genes whose expression was regulated by KLF5 in the mouse models are shown in Table S3. To obtain KLF5-IS, the signature scores were calculated as the ratio of the mean expression (normalized TPM) of CP and CI signature genes. A total of 360 TNBC patients were then stratified based on the level of KLF5-IS scores, and survival curves were generated using the Kaplan‒Meier method and the survival package. We used log-rank test statistics to assess the significance between groups.

### Statistical analysis

Statistical analyses were performed using GraphPad Prism version 8.0. All experiments were performed at least three times independently. The results are presented as means ± SD. The relative increase in protein expression was quantified using ImageJ software and was normalized to control protein expression in each experiment. Datasets obtained from different experimental conditions were compared with t test when comparing only 2 groups. Multiple comparisons between groups were performed using the Mann-Whitney U test or Tukey's multiple comparison test. Survival probabilities for recurrence-free survival (RFS) were estimated using the Kaplan-Meier method, and variables were compared using the log-rank test. Pearson's correlation was used to evaluate the correlations. In the bar graphs, a single asterisk (*) indicated p < 0.05, two asterisks (**) indicated p < 0.01, and three asterisks (***) indicated p < 0.001.

## Results

### The deficiency of Klf5 in tumors decelerates tumor progression depending in part on the functions of Cd8^+^ T cells

**Figure 1:**
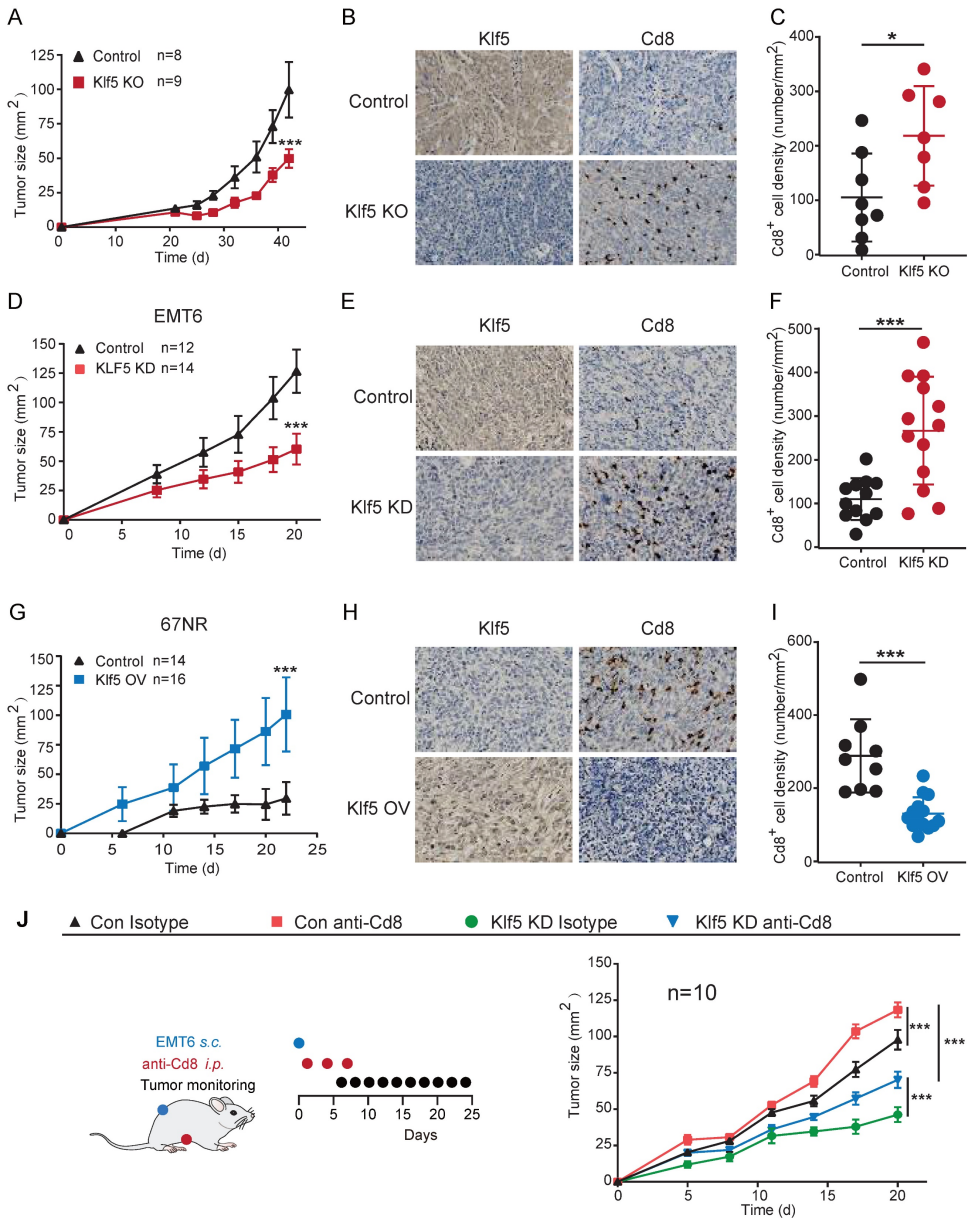
** KLF5-mediated tumor growth partially depends on CD8+ T lymphocytes.** (**A, D and G**) Tumor growth curves of mice inoculated with 5 × 10^5^ PyMT/ PyMT-cre (Klf5 KO)-induced tumor cells (**A**), 3 × 10^5^ con or Klf5 knockdown (KD) EMT6 (**D**), or 4 × 10^6^ con or Klf5 overexpression (OV) 67NR (G). (**B-C, E-F and H-I**) The levels of Klf5 and Cd8 were quantified by ImageJ after staining with specific antibodies in paraffin-embedded tissues obtained from murine tumors. Representative images of Klf5 and Cd8 (**B, E and H**). The level of Cd8^+^cells was quantified in (**C, F and I**). Scale bar equals 50 μm. (**J**) Tumor growth curves of BALB/c mice inoculated with con or Klf5 KD EMT6 cells (3 × 10^5^) treated with isotype control or depleting anti-Cd8 antibodies. n ≥ 8 for mice in each group. Tumor growth curves (mean ± SEM) and Cd8 expression (mean ± SD) were plotted (*p < 0.05 or ns, not statistically significant vs. control; two-way ANOVA or Student's t test).

To explore whether Klf5 contributes to tumorigenesis, we developed a murine breast cancer model with Klf5 knockout (KO)^15^. FVB/N Klf5-LOXP mice at 5 weeks of age were intraductally injected with lentiviruses carrying polyoma middle T-antigen (PyMT) and PyMT-Cre. Next, these tumors were isolated, dissociated and cultivated. The Klf5 level in tumor cells was verified by WB (**Figure S1C**). Subsequently, neoplastic cells with or without Klf5 expression were seeded into the mammary fat pads of FVB/N mice (**Figure S1A**). Tumors with Klf5 knockout grew more slowly than those in the control group (**Figures 1A and S1D-E**). Furthermore, we screened Klf5 expression in multiple murine cancer cells, showing that Klf5 was highly expressed in murine breast cancer EMT6 cells and murine colon cancer CT26 cells but expressed at low levels in 67NR cells (murine breast cancer cells) (**Figure S1B**). We generated EMT6 cells with Klf5 knockdown (KD) and 67NR cells overexpressing Klf5 (**Figure S1F, I**). We next measured tumor growth in immunocompetent BALB/c mice by injection of different cells. Depletion of Klf5 in EMT6 cells substantially retarded tumor growth (**Figure 1D and S1F-H**), while tumors derived from 67NR cells overexpressing Klf5 grew faster than those from control cells (**Figure 1G and S1I-K**).

To investigate whether Klf5-modulated tumor growth was mediated by Cd8^+^ T cells, the infiltrating level of Cd8^+^ T cells was detected in the abovementioned mouse models by immunohistochemistry. A marked increase in Cd8^+^ T cells was found in tumors with Klf5 KO or KD compared with those in the control group. By contrast, tumors carrying cells with Klf5 overexpression showed reduced Cd8^+^ T-cell infiltration (**Figure 1B-C, E-F and H-I**). Furthermore, an anti-Cd8 neutralizing antibody was applied to block Cd8^+^ T cells in the EMT6 mouse model, indicating that Cd8^+^ T-cell depletion facilitated tumor growth in both the control and Klf5 KD groups (**Figure 1J and S1L-M**). Taken together, the results demonstrated that Klf5 contributes to accelerating tumor growth partly by impairing the infiltration of Cd8^+^ T cells.

### Transcriptome profiling reveals that Klf5 regulates the tumor immune microenvironment

**Figure 2:**
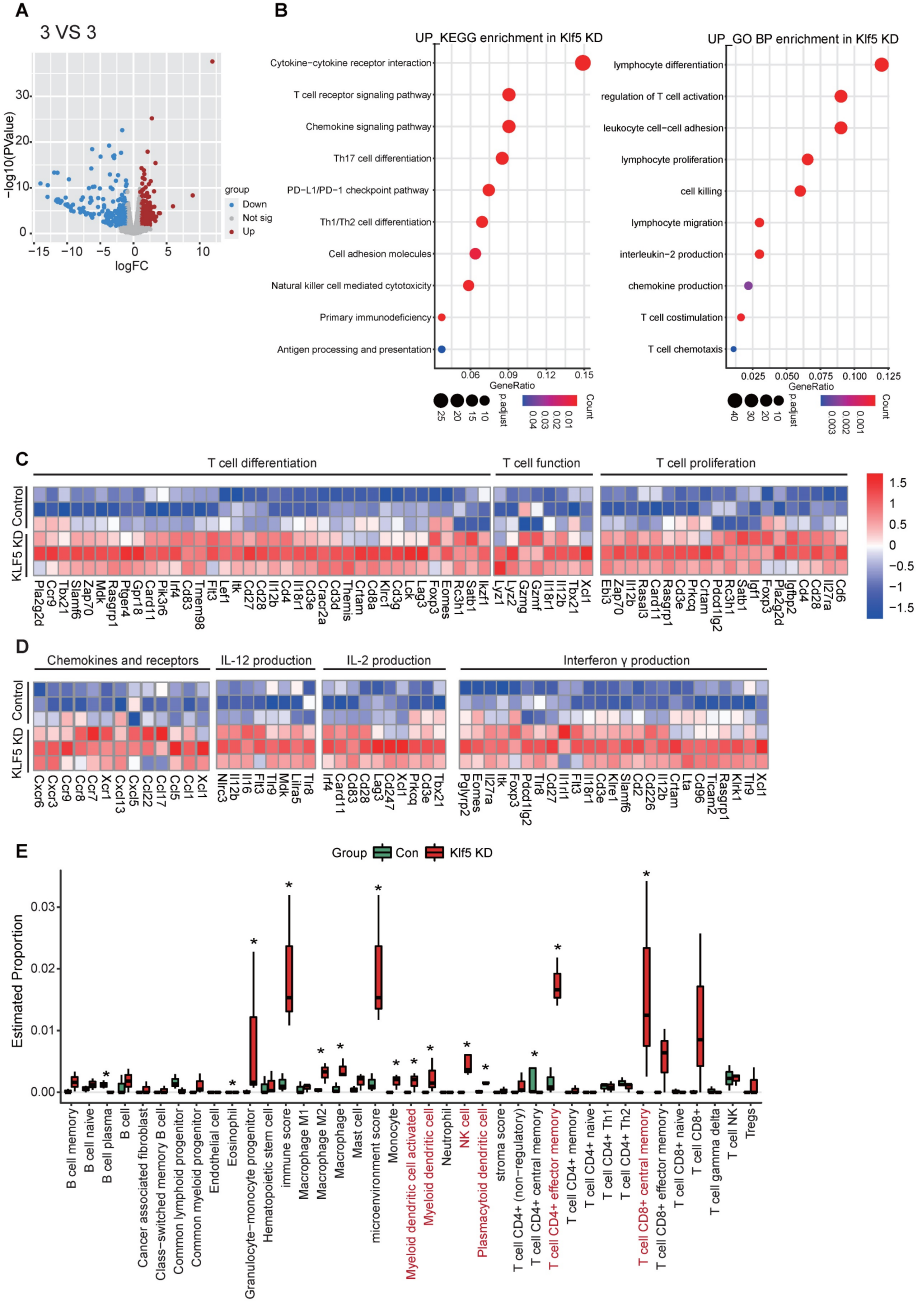
** Loss of Klf5 is associated with an immunostimulatory microenvironment.** BALB/c mice were inoculated with control or Klf5 KD EMT6 cells. When tumors grew for approximately 14 days, they were collected for RNA transcriptional sequencing (3 VS 3). (**A**) Volcano plot of differentially expressed genes in Klf5-deficient versus control EMT6 tumors. Significant gene expression with changes > 1.5-fold and P <0.05 was considered. (**B**) Enrichment of KEGG signature scores and Gene Ontology (GO) analysis were performed in transcriptional profiles for Klf5 KD vs. control groups. (**C**) Heatmap of the differentially expressed genes associated with T-cell proliferation, differentiation, chemotaxis and function in Klf5-deficient versus control EMT6 tumors. (**D**) The Xcell method was performed to define the immune cell populations in Klf5-deficient versus control EMT6 tumors. Significantly activated molecules or cell populations are highlighted in red. *p < 0.05.

To evaluate whether Klf5 alters the tumor-immune microenvironment, we performed RNA-sequence analysis on tumor tissues from EMT6 or 67NR mouse tumor models. Significant gene expression with changes > 1.5-fold and p < 0.05 was considered (**Figure 2A and S2A**). To characterize the biological processes underlying the transcriptional changes in control tumor tissues and Klf5 KD tumor tissues, KEGG signature scores and Gene Ontology (GO) analysis were performed. A striking enrichment of T-cell proliferation, differentiation, chemotaxis and activation and other antitumor immune pathways was observed in tumors with low Klf5 expression (**Figure 2B-C**). Among the profoundly upregulated genes in the Klf5 KD group, most were associated with T-cell differentiation (such as *Eomes*, *Irf4* and *Foxp3*), proliferation (such as *Cd28*), chemotaxis (such as *Ccl5*, *Ccr7*, *Ccr9*, *Cxcr3* and *Cxcr6*) and activation, including Il-12, Il-2 and interferon γ (Ifnγ) production and *Gzmg* and *Gzmf* overexpression (**Figure 2D**). Subsequently, we deduced the cell composition in tumors from the control and Klf5 KD groups via the X-cell method. The analysis demonstrated that naïve and activated dendritic cells (DCs), NK cells, Cd4^+^ effector memory T cells and Cd8^+^ effector memory T cells were enriched within tumors carrying Klf5 KD cells (**Figure 2E**). Conversely, the tumors from 67NR overexpressing Klf5 were enriched in multiple pathways involving immune functions: “lymphocyte migration”, “chemokine-mediated signaling pathway” and “lymphocyte chemotaxis” (**Figure S2B**). Likewise, gene expression analysis revealed a profound increase in immunosuppressive markers (such as *Cxcl1* and *Il10*) but a decrease in immune-supporting genes (such as *Tnfrsf19*, *Tnfrsf18*, *Cxcr3*, *Cx3cr1* and *Cxcl13*) (Figure S2C). Concerning cell composition in the TME, a reduction in the number of naïve and activated dendritic cells (DCs), naïve Cd8^+^ T cells, Cd4^+^ effector memory T cells, Cd8^+^ effector memory T cells and in the total Immunoscore was found in tumors with Klf5 overexpression (**Figure S2D**). Thus, tumor-intrinsic Klf5 expression may contribute to the alteration of overall immune compositions in the TME by mediating the proliferation, differentiation, chemotaxis and activation of T cells.

### KLF5 promotes PGE2 production by augmenting* COX2* gene transcription

**Figure 3:**
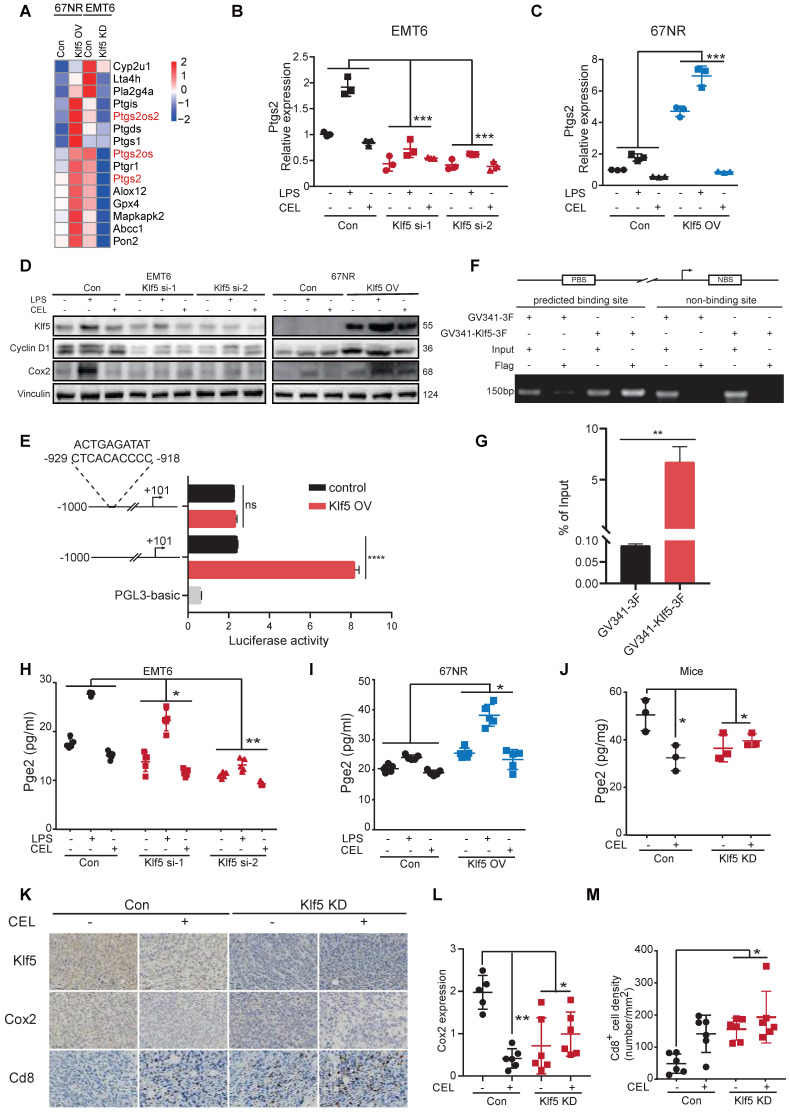
** Klf5 is necessary for tumor-intrinsic PGE2 generation.** (**A**) Heatmap of differentially expressed genes involved in arachidonic acid metabolism from both Klf5-deficient or control EMT6 and Klf5-overexpressing or control 67NR tumors. (**B**) Relative Ptgs2 (encoded COX2) mRNA expression in control and Klf5-deficient EMT6 cells in the presence or absence of LPS (1 μg/mL) or celecoxib (CEL) (50 μM). (**C**) Relative Ptgs2 (encoded COX2) mRNA expression in control and Klf5-overexpressing 67NR cells treated with or without LPS (1 μg/mL) or celecoxib (CEL) (50 μM). (**D**) Western blot analysis of Klf5, CyclinD1 and Cox2 in both Klf5-deficient or control EMT6 and Klf5-overexpressing or control 67NR cells following treatment with or without LPS (1 μg/mL) or CEL (50 μM) for 24 h. (**E**) HEK293T cells were cotransfected with Ptgs2 (-1000/+101)-luc or Ptgs2mut (-1000/+101)- luc plus GV341-KLF5 or control vector GV341 and the internal control plasmid pRL-TK. (**F-G**) 67NR wt/Klf5-3F-OV cells were subjected to ChIP assays using anti-Flag magnetic beads. PCR was performed to amplify regions surrounding the putative Klf5 binding region and a nonspecific Klf5 binding region. (**H-I**) The secreted levels of PGE2 were detected by ELISA in both Klf5-deficient or control EMT6 and Klf5-overexpressing or control 67NR cells following treatment with or without LPS (1 μg/mL) or CEL (50 μM) for 24 h. (**J**) Pge2 levels were evaluated in Klf5-deficient versus control EMT6 tumors receiving daily CEL (30 mg/kg) treatment for 1 week (n = 3). (**K-M**) The Klf5, Cox2 and Cd8 levels were quantified by ImageJ after staining with specific antibodies in paraffin-embedded tissues obtained from Klf5-deficient versus control EMT6 tumors. Representative images of Klf5, Cox2 and Cd8 (**K**). Cox2 expression was quantified in (**L**). The level of Cd8^+^ cells was quantified in (**M**). Scale bar equals 50 μm. The data are represented as means ± SD. n ≥ 5 for mice in each group. (*p < 0.05; **, p < 0.01; ***, p < 0.001 significant vs. control; one-way or two-way ANOVA).

Our previous study revealed that KLF5 promoted PGE2 production in TNBC by inducing *mPGES1* transcription 14. We reanalyzed the transcription profiles mentioned above and found that hallmarks of the arachidonic acid catabolic pathway were substantially changed in tumor tissues. Interestingly, *Ptgs2* (encoding Cox2 protein) expression was positively related to the Klf5 levels (**Figure 3A**). To further verify whether KLF5 promotes COX2 expression, EMT6 and 67NR cells were treated with lipopolysaccharide (LPS, an inducer of COX2 expression 38) or COX2 inhibitor (celecoxib, CEL). Klf5 KD by siRNA silencing decreased *Ptgs2* mRNA and protein expression, while ectopic Klf5 overexpression profoundly upregulated *Ptgs2* mRNA and protein (**Figure 3B-D**). Consistently, LPS failed to induce Cox2 expression after Klf5 was knocked down. By contrast, Klf5 overexpression amplified the effect of LPS-induced Cox2 expression (**Figure 3C-D**). We also verified that Klf5 positively regulated mPegs1 expression in EMT6 and 67NR cells (**Figure S3E-G**). The KLF5 transcription factor regulates *PTGS2* mRNA transcription through the PTGS2 promoter. To test this, we found several potential KLF5 binding sites on website tools and after a review of the literature 39. Next, we generated luciferase reporter constructs by cloning the *PTGS2* gene promoter (mouse: -1000/+101; human: -1100/+100) into the PGL3-BASIC plasmid. Dual luciferase assays in HEK293T cells found that the luciferase reporter constructs were significantly activated by KLF5 (**Figure 3E; Figure S3B**). To further validate whether the predicted KLF5 binding site is responsible for KLF5-mediated transcriptional activation, we mutated the predicted binding site. Indeed, the mutation completely abrogated KLF5-mediated *PTGS2* gene promoter activation in HEK293T cells (**Figure 3E; Figure S3B**), confirming that the putative KLF5 binding site is necessary for *PTGS2* gene promoter activation by KLF5. Finally, we demonstrated that Klf5 binds to the *Ptgs2* gene promoter using chromatin immunoprecipitation (ChIP) assays in 67NR wt/Klf5-3F OV cells (**Figure 3F-G**). Consistently, only the anti-KLF5 antibody, but not the control goat IgG, specifically immunoprecipitated the promoter in HCC1806 cells (**Figure S3C-D**). As expected, depletion of the Klf5 or COX2 inhibitor observably reduced PGE2 levels *in vitro* and *in vivo*. Conversely, KLF5 upregulation and LPS stimulated PGE2 secretion (**Figure 3H-J**). Finally, we detected Cox2 expression and Cd8^+^ T-cell infiltration in mice inoculated with control or Klf5 KD tumor cells in the presence or absence of CEL. For this analysis, the Cox2 levels in tumors carrying Klf5 KD tumor cells were lower than those in the control group. Conversely, Cd8^+^ T cells were significantly more abundant in the Klf5 KD group than in the control group (**Figure 3K-M**). Therefore, KLF5 facilitates *COX2* and *mPGES1* transcription to increase PGE2 production and decrease CD8^+^ T cell infiltration.

### Inhibition of the Klf5/Cox2 axis increases the number and functionality of intratumoral antitumor T cells

**Figure 4:**
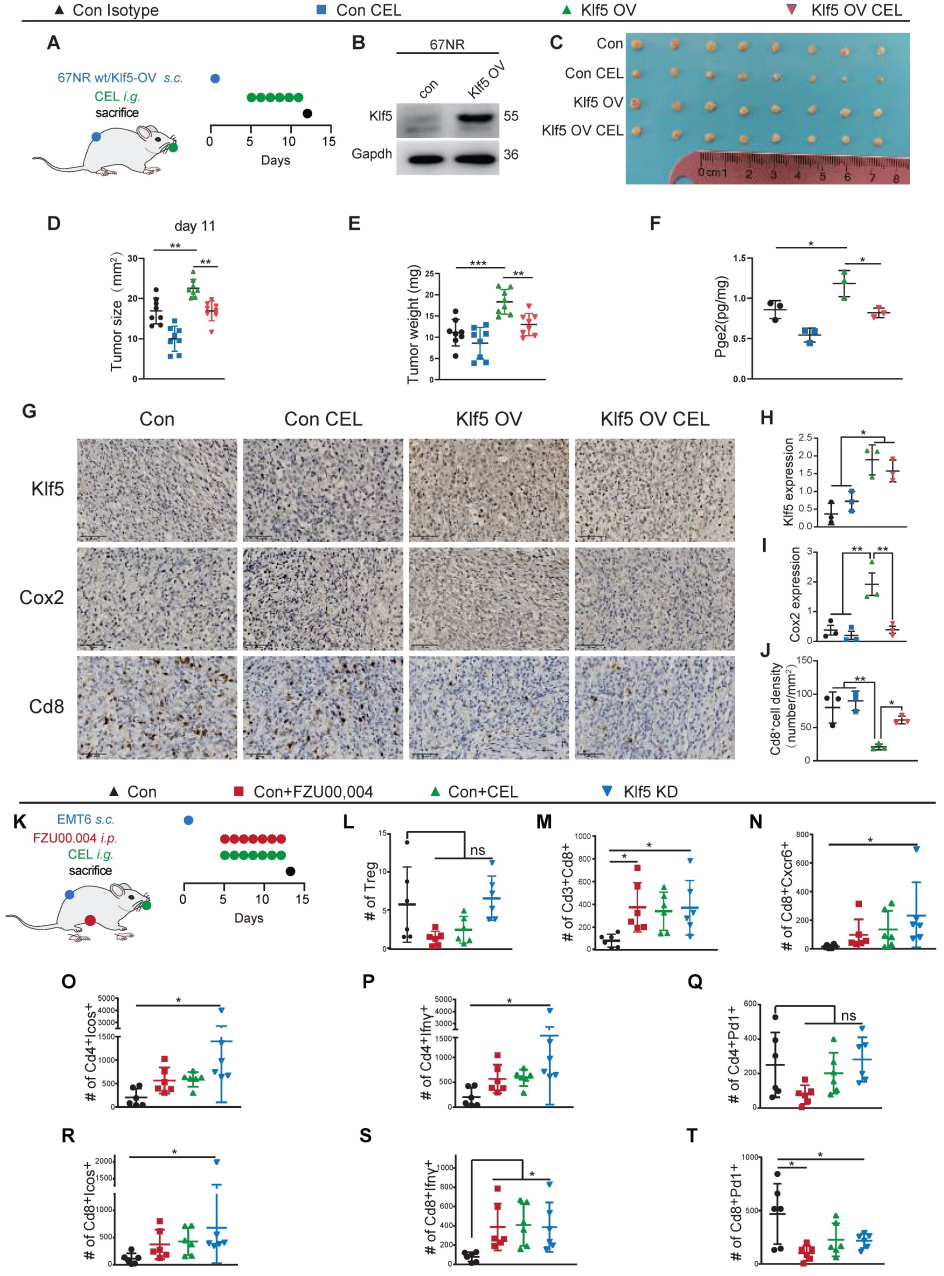
** Inhibition of the Klf5/Cox2 axis increases the number and functionality of antineoplastic T lymphocytes in tumors.** (**A-B**) Mice inoculated with control or Klf5-overexpression 67NR cells were treated every day with COX2 inhibitor (CEL) (total 6 times) when tumors were approximately 20 mm^2^ in mean area. (**C-E**) Tumor size and weight distributions at Day 11 are shown. (**F**) Pge2 levels were evaluated in Klf5-overexpression versus control 67NR tumors receiving daily CEL (30 mg/kg) treatment for 6 days (n = 3). (**G-J**) The Klf5, Cox2 and Cd8 levels were quantified by ImageJ after staining with specific antibodies in paraffin-embedded tissues obtained from Klf5- overexpression versus control 67NR tumors. Representative images of Klf5, Cox2 and Cd8 (G). Klf5 expression was quantified in (**H**). Cox2 expression was quantified in (**I**). The level of Cd8^+^cells was quantified in (**J**). (**K**) Schematic of the experimental setup. Cytofluorometric analysis of tumor-infiltrating lymphocytes (TIL): Cd4^+^Foxp3^+^Cd25^+^ regulatory T cells (Treg) (**L**), Cd3^+^Cd8^+^ cytotoxic T lymphocytes (**M**), Cd8^+^Cxcr6^+^ T lymphocytes (N), and quantification of Icos, Ifnγ and Pd1 expression among both Cd4^+^ and Cd8^+^ T cells (**O-T**). Scale bar equals 50 μm. The data are represented as means ± SD. n ≥ 3 for mice in each group. (*p < 0.05; **, p <0.01; ***, p <0.001 significant vs. control; one-way or two-way ANOVA).

To address the necessity of Klf5/Cox2 axis for tumor immunity regulation, we developed subcutaneous tumor mouse models with 67NR wt/Klf5-OV cells (**Figure 4A-B**). Klf5 overexpression induced pro-tumorigenic effect and Pge2 enhancement should be partially reversed by COX2 inhibitor (CEL) *in vivo* (**Figure 4C-F**). Additionally, Klf5/Cox2 axis activation reduced the number of tumor-infiltrating CD8^+^ T cells, and CEL partially increased CD8^+^ T cell infiltration (**Figure 4G-J**). To decipher the Klf5-mediated alteration of the immune landscape in the TME, multicolor flow cytometry was performed to profile the infiltrating immune cell components in the TME (**Figure 4K**). Genetic ablation of Klf5, FZU00,004 (a KLF5 inhibitor) and CEL failed to reduce the frequency of tumor-infiltrating Tregs (Cd4^+^Cd25^+^Foxp3^+^ T cells), but Klf5 depletion increased the number of Cd3^+^Cd8^+^ T cells and increased the Cd3^+^Cd8^+^/Treg ratio (**Figure 4L-N**). The proliferation and function of T cells were further examined. Inducible costimulator (ICOS) is a conserved marker of proliferated T cells 40. Klf5 silencing led to marked augmentation of Icos-positive populations in both Cd4^+^ and Cd8^+^ T cells (**Figure 4O, R**). Regarding T-cell functionality, Klf5 knockdown in tumors facilitated Cd4^+^ T cells to secrete interferon gamma (Ifnγ), while blockade of the Klf5/Cox2 axis enhanced the Ifnγ release of Cd8^+^ T cells (**Figure 4P, S**). Additionally, we detected the number of Pd1^+^ cells, showing that Klf5 deficiency resulted in a reduced number of Cd8^+^Pd1^+^ T cells but not Cd4^+^ T cells (**Figure 4Q, T**). In the transcription profile, we observed increased Cxcr6 expression of Klf5 KD tumors. Cxcr6 is a classical biomarker of resident memory CD8^+^ T (Trm) cells to sustain tumor control 41, 42, and our results demonstrated that Klf5 deletion specifically promoted the infiltration of Cd8^+^Cxcr6^+^ T cells in tumors (**Figure 4N**). Thus, blocking the Klf5/Cox2 pathway within cancer cells may increase the quantity and activity of antineoplastic T-cell populations, causing the expansion of Trm cells, which protect against tumorigenesis.

### Blocking the Klf5/Cox2 pathway synergizes with the antitumorigenic effects of anti-Pd1 therapy

**Figure 5:**
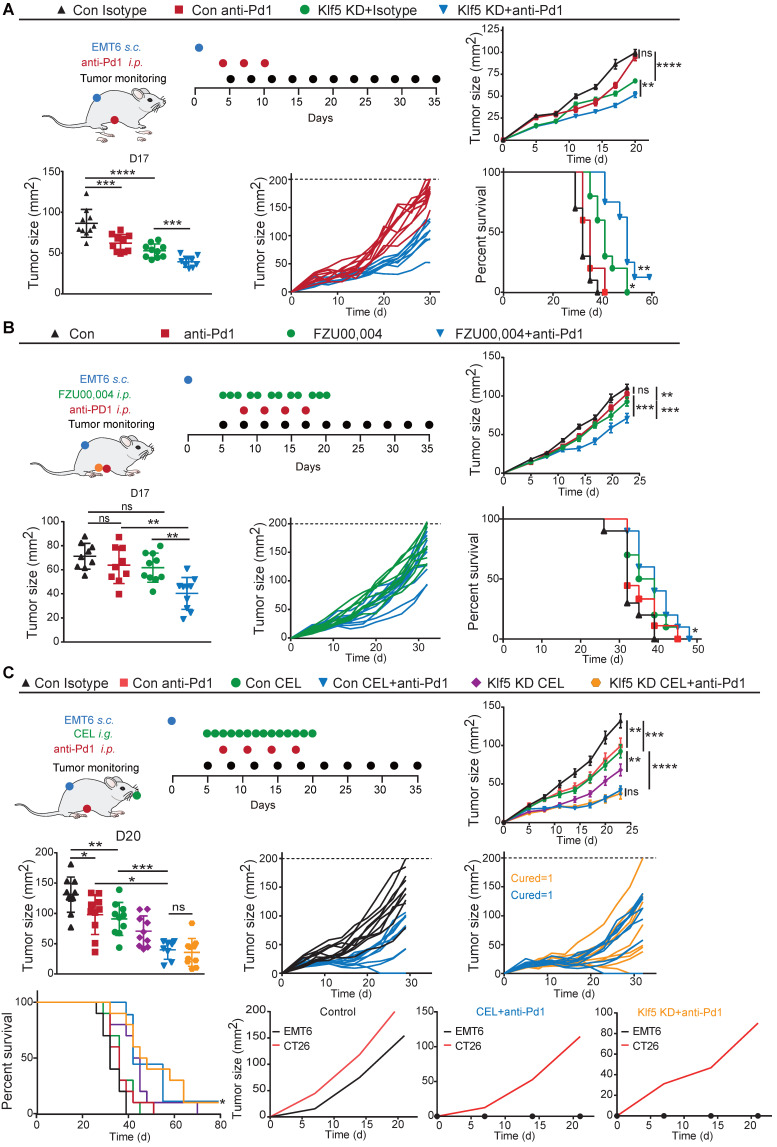
** Blocking the Klf5/Cox2 axis potentiates the antitumorigenic effects of anti-PD1 blockade.** (**A**) Mice inoculated with control or Klf5-deficient EMT6 cells were treated every three days with anti-Pd1 blocker (total 3 times) when tumors were approximately 20 mm^2^ in mean area. Growth curves (mean ± SEM), tumor size distributions at Day 17, individual tumor growth curves and survival curves are shown. (**B**) Mice inoculated with control EMT6 cells were continuously treated for three or two days with FZU00,004 for two weeks with or without an anti-Pd1 blocker when tumors were approximately 20 mm^2^ in mean area. Growth curves (mean ± SEM), tumor size distributions at Day 17, individual tumor growth curves of mice and survival curves are shown. (**C**) Mice inoculated with control EMT6 cells were continuously treated with CEL for two weeks with or without an anti-Pd1 blocker when tumors were approximately 20 mm^2^ in mean area. Growth curves (mean ± SEM), tumor size distributions at Day 20, individual tumor growth curves and survival curves are shown. n ≥ 8 for mice in each group. (*p < 0.05; **, p <0.01; ***, p <0.001; ****, p <0.0001 or ns, not statistically significant vs. control; two-way ANOVA). The generation of immunological memory was assessed in cured animals by rechallenge with EMT6 and CT26. A fresh mouse (n = 1) was synchronously challenged with EMT6 and CT26 as a control.

To investigate whether inhibition of the Klf5/Cox2 axis reinforces the efficiency of immune checkpoint blockade and considering the high expression of Klf5 in EMT6 and CT26 cell lines, ablation of cancer cell-intrinsic Klf5 in the CT26 colon and EMT6 breast cancer models was first applied to test the hypothesis. Mice with EMT6 or CT26 tumors were unresponsive to anti-Pd1 monotherapy, while the anti-Pd1 blocker resulted in obvious tumor regression and prolonged survival in mice with Klf5 KD tumors (**Figures 5A and S4A**). Furthermore, we combined FZU00,004 or celecoxib and an anti-Pd1 inhibitor in murine tumor models. Monotherapy with FZU00,004 or celecoxib could moderately reduce tumor growth, whereas the combination markedly controlled tumor growth, resulting in tumor eradication in several cases and increased overall survival in two tumor models (**Figure 5B-C and S4A**). Additionally, these mice inoculated with control or Klf5-deficient EMT6 cells experienced complete tumor remission, then they were rechallenged with EMT6 cells or CT26 cells, and they were resistant against EMT6 cells but facilely developed CT26 tumors, suggesting that they formed immune memory (**Figure 5C**). These results highlight that Klf5/Cox2 blockade can potentiate the efficacy of immune-targeting drugs in preclinical models.

### Single-cell and spatial analyses decipher KLF5-mediated alterations in tumor-infiltrating immune compartments

**Figure 6:**
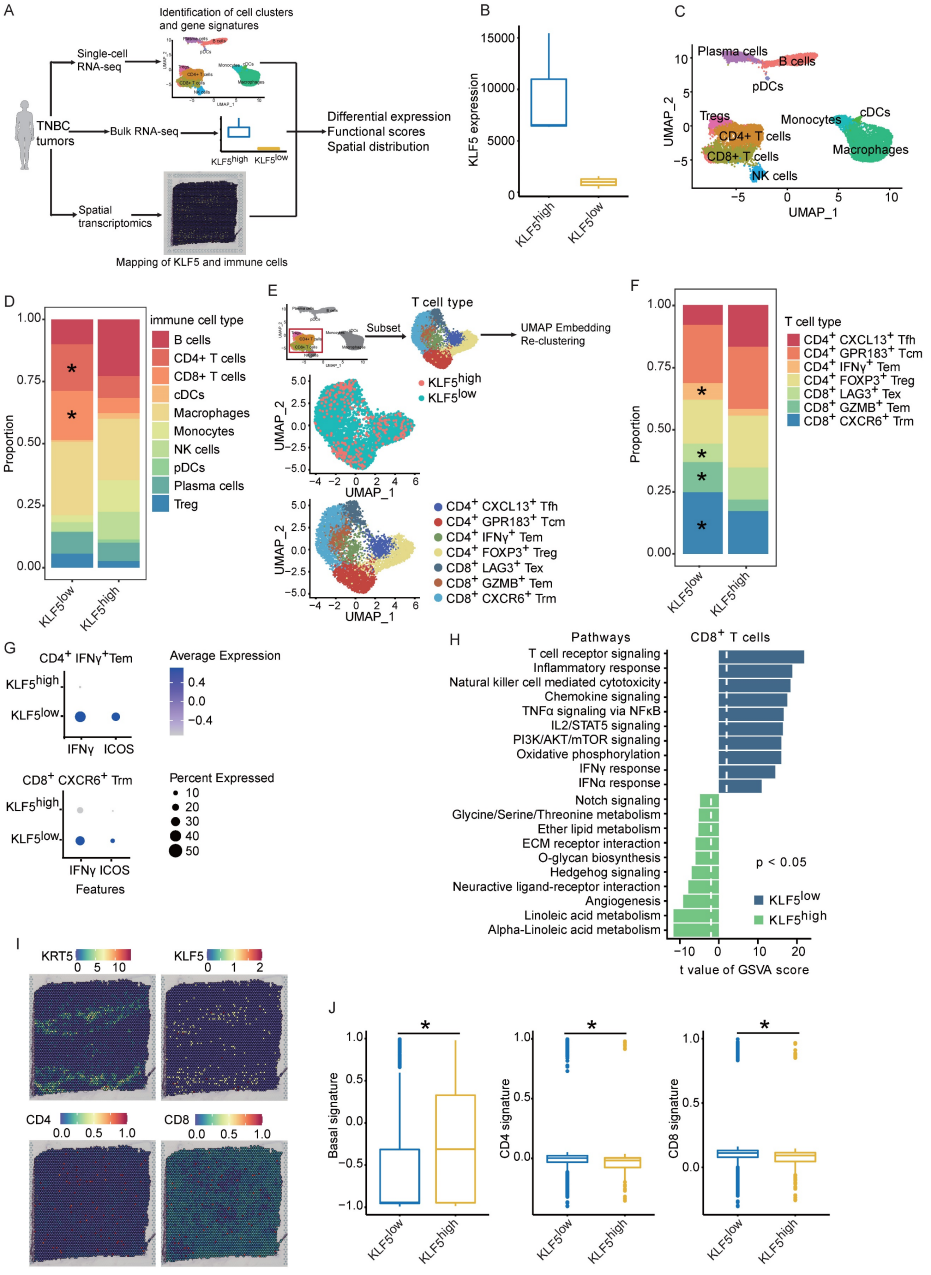
** Single-cell and spatial analyses reveal KLF5-associated remodeling of the tumor immune infiltrate.** (**A**) Schematic of single-cell, bulk RNA-seq and spatial RNA-seq experiments and analyses. (**B**) Six TNBC samples were divided into two subgroups based on KLF5 expression in bulk RNA-seq. (**C**) Identification of tumor-infiltrating immune cell populations. Uniform manifold approximation and projection (UMAP) embeddings of single-cell RNA-seq profiles from 9,104 CD45^+^ leukocyte cells showing 10 clusters identified by integrated analysis, colored by cluster. (**D**) Bar plot of proportional differences in immune cells between the KLF5^high^ and KLF5^low^ groups. (**E**) Reclustering of T lymphocytes, UMAP visualization and marker-based annotation of 2 KLF5 groups and 8 T lymphocyte subtypes, colored by cluster identity. (**F**) Bar plot of proportional differences in T lymphocytes between the KLF5^high^ and KLF5^low^ groups. (**G**) Bubble heatmap of functional analysis of CD4^+^IFNγ^+^ Tem and CD8^+^CXCR6^+^Trm cells. The dot size indicates the fraction of expressing cells, colored based on normalized expression levels. (**H**) Enrichment of different gene signature scores altered by KLF5 expression levels in single-cell transcriptomes from reclustered CD8^+^ T cells. (**I**) Different spatial distributions of KLF5 and T lymphocyte subpopulations were overlaid onto tissue spots. (**J**) Box plots show the enrichment scores of the basal signature, CD4 signature and CD8 signature in KLF5^high^ and KLF5^low^ regions.

To evaluate whether KLF5 alters the human (TIME), we reanalyzed our previous and public datasets, including scRNA-seq, bulk RNA-seq and spatial transcriptome (ST)^43^, ^44^. In the public dataset, 6 TNBC samples were simultaneously detected by scRNA-seq and bulk RNA-seq. These samples were divided into two groups (3 for each group) based on KLF5 expression in bulk RNA-seq (**Figure 6A-B**). First, we performed unsupervised clustering analysis on integrated single-cell profiles from KLF5^low^ and KLF5^high^ tumors to define major immune cell clusters. A total of 10 distinct clusters were annotated based on the expression of classic biomarkers (**Figure 6C and S5A**).

Compared with the number in KLF5^high^ tumors, the number of CD4^+^ and CD8^+^ T lymphocytes was significantly increased in samples from KLF5^low^ tumors, whereas the percentage of monocytes was markedly reduced (**Figure 6D**). Furthermore, we reclustered T cells into several subpopulations, and seven subsets were identified (**Figure 6E**). T lymphocyte subpopulations were primarily defined as CD8^+^ or CD4^+^ T cells. CD4^+^ T lymphocytes were identified as follicular helper T cells (Tfh), central memory T cells (Tcm), effector memory T cells (Tem) and regulatory T cells (Treg) based on the expression of the corresponding markers (**Figure 6E and S5B**). Likewise, CD8^+^ T lymphocytes were characterized as exhausted T cells (Tex), effector memory T cells (Tem) and tissue-resident memory T cells (Trm) based on the classical markers (**Figure 6E and S5B**) 37. The proportions of CD4^+^IFNγ^+^ Tem, CD8^+^GZMB^+^ Tem and CD8^+^CXCR6^+^ Trm cells were dramatically upregulated in KLF5^low^ tumors, while the relative ratio of CD8^+^LAG3^+^ Tex cells was low (**Figure 6F**). When we performed functional analysis of CD4^+^IFNγ^+^ Tem cells and CD8^+^CXCR6^+^ Trm cells, the genes (*ICOS* and *IFNγ*) involved in the proliferation and function of effector T cells were enriched in KLF5^low^ cells (**Figure 6G**). Additionally, we scored the gene signatures within all CD8^+^ T lymphocytes, revealing that T-cell receptor signaling, the IFNα response, the IFNγ response, oxidative phosphorylation and PI3K/AKT/mTOR signaling were enriched in KLF5^low^ tumors; by contrast, o-glycan biosynthesis, angiogenesis and linoleic acid metabolism were enriched in KLF5^high^ samples (**Figure 6H**). In addition, we explored whether KLF5 affects the spatial distribution of T lymphocytes. ST analysis was performed to map the location of KLF5, CD4^+^ T cells and CD8^+^ T cells. KLF5 was coexpressed with basal markers (KRT5, KRT14 and KRT17) in breast cancer tissue. By contrast, CD4^+^ and CD8^+^ T lymphocytes spatially prevailed in KLF5^low^ regions (**Figure 6I-J and S5E-F**). The expression of these biomarkers in the ST sample was also validated by immunohistochemistry (IHC), and similar results were observed (**Figure S5D**). Taken together, the results demonstrated that both CD4^+^ and CD8^+^ T lymphocytes were abundant in KLF5^low^ tumors and displayed enhanced proliferation and functionality.

### A KLF5-associated immune gene score exhibits independent prognostic utility

**Figure 7:**
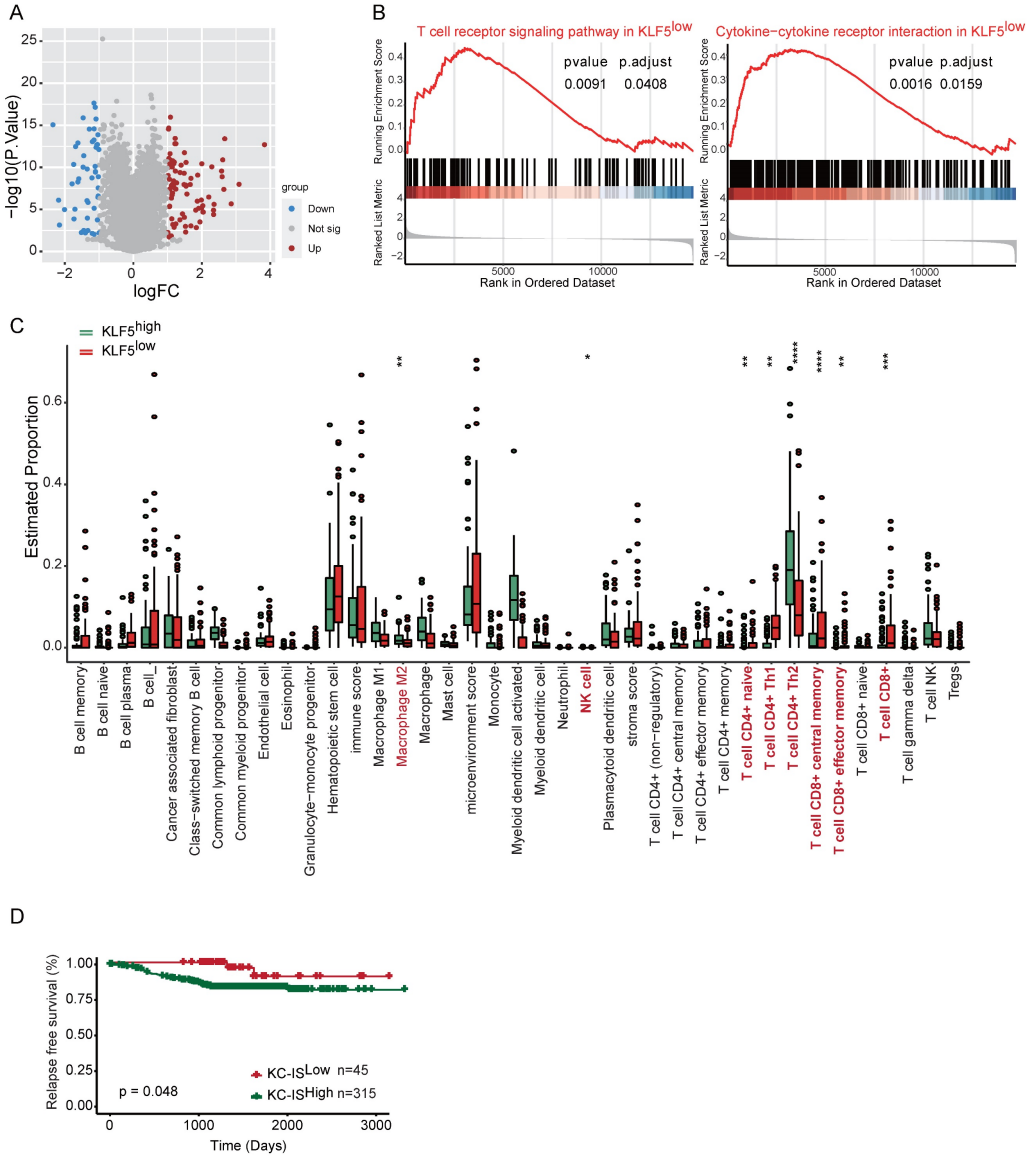
** The KLF5-associated immune gene score exhibits independent prognostic utility in BLBC.** The transcriptional profiles of BLBC with low TGFβ expression (n = 180) were obtained from the FUSCC cohort. Based on the expression of KLF5, the cohort was divided into KLF5^low^ and KLF5^high^ groups. (**A**) Volcano plot of differentially expressed genes in KLF5^low^ and KLF5^high^ samples. Significant gene expression with changes > 1.5-fold and P <0.05 was considered. (**B**) GSEA was performed to estimate the biological processes in transcriptional profiles for KLF5^low^ vs. KLF5^high^ groups. (**C**) The Xcell method was performed to define the immune cell populations in KLF5^low^ and KLF5^high^ cases. Significantly activated molecules or cell populations are highlighted in red. (Student's t test). (**D**) Survival analysis of BLBC patients stratified according to the KC-IS. Kaplan‒Meier survival plots parsed as high versus low on a median cutoff for KC-IS.

To explore whether the molecular features of the KLF5/COX2-driven immune microenvironment exist in human BLBC, we assessed transcriptomic profiles from Fudan University Shanghai Cancer Center (FUSCC)^45^-^47^. TGFβ has been found to inhibit KLF5-induced protumor activity 25, 48; consequently, cases with low TGFβ expression in FUSCC were collected for further analysis. First, we examined the expression of KLF5 and COX2 in the subtypes of TNBC, revealing that both KLF5 and COX2 were highly expressed in the BLBC subpopulation (**Figure S6A**). Next, the distinct gene expression between KLF5 low expression and high expression with changes > 1.5-fold and p <0.05 was considered (**Figure 7A**). GSEA and GO analysis were performed to evaluate the biological processes based on the transcriptional changes in the samples with low and high levels of KLF5. Several immune-associated pathways, including “T-cell receptor signaling”, “cytokine‒cytokine receptor interaction” and “negative regulation of T-cell apoptosis”, were enriched in the KLF5^low^ group (**Figure 7B and Figure S6B**). Additionally, bioinformatic analysis of immune cell composition demonstrated that intratumoral NK cells, naïve CD4^+^ T cells, CD4^+^ T helper 1 (Th1) cells, total CD8^+^ T cells, CD8^+^ central memory cells and effector memory T cells were abundant in the KLF5^low^ group, while M2 macrophages and CD4^+^ T helper 2 (Th2) cells were positively associated with KLF5 expression (**Figure 7C**). To assess the prognostic value of KLF5/COX2-driven immune profiles, we further generated a KLF5/COX2-associated immune score (KC-IS) based on the integration of KLF5/COX2-mediated immune genes (**Table S3**). The BLBC patients were stratified according to the KC-IS, showing that patients with high KC-IS exhibited a poor prognosis (**Figure 7D**). Similarly, in the colon cohort, KLF5^+^/CD8^-^ was associated with poor survival (**Figure S6C-D**). In summary, KC-IS is a potent indicator of the outcome in BLBC and colon cancer.

## Discussion

Given that the KLF5 transcription factor promotes tumor proliferation, invasion and stemness in diverse cancers 9, its role in antitumor immunity remains largely unknown. In the present study, KLF5 deficiency impeded breast tumor growth by increasing the infiltration and functionality of antineoplastic T cells. Mechanistically, KLF5 modulates PGE2 production by transcriptionally activating COX2. Genetic or pharmacological inactivation of the KLF5/COX2 axis develops an immune-supportive microenvironment and sensitizes tumors to anti-PD1 therapy. In single-cell analysis, low expression of KLF5 was positively correlated with enrichment of CD4^+^IFNγ^+^ Tem, CD8^+^GZMB^+^ Tem and CD8^+^CXCR6^+^ Trm cells. Importantly, KLF5/COX2-mediated immune profiles display prognostic value in breast and colon cancer.

Accumulating evidence has shown that KLF5 may remodel the tumor microenvironment. In our results, genetic ablation of KLF5 not only expedites the proliferation and function of both CD4^+^ and CD8^+^ T cells but also induces the accumulation of Cxcr6^+^ Trm cells in tumors. CXCR6 was highly expressed in CD8^+^ T cells and was considered a typical marker of Trm cells 41, 42. Trm cells extensively spread over the liver, lung, intestine and regional lymph nodes. In the TME, CCR7^+^ dendritic cells recruit CXCR6^+^ Trm cells by releasing the CXCR6 ligand CXCL16^42^. ICOS stimulation hinged the optimal production of Trm cells 49. Additionally, our results showed that Klf5 deletion contributed to a profound increase in CD8^+^ICOS^+^ T cells, which may cause the accumulation of Trm cells in KLF5-deficient tumors. Functionally, CXCR6^+^ Trm cells are required to sustain the proliferation and antitumor effects of cytotoxic T lymphocytes 42, 50. CXCR6^+^ Trms control tumor growth and metastasis 42, 50, 51 and are equipped with immunosurveillance to restrain tumor recurrence 52, 53. A recent study demonstrated that Klf5 loss led to a reduced number of myeloid-derived cells, particularly granulocytic myeloid-derived suppressor cells (gMDSCs), but an augmented number of both CD4^+^ and CD8^+^ T cells in pancreatic cancer models 26. However, the molecular mechanisms were not completely addressed. First, cancer stem cells (CSCs) were found to mediate tumor immune evasion. These CSCs secrete chemokines such as CCL1 and CCL5 to recruit MDSCs; in turn, MDSCs support CSC proliferation 54. Notably, KLF5 is a key transcription factor that maintains tumor stemness 9, suggesting that KLF5 may impair antitumor immunity through the sustainability of neoplastic stemness. Additionally, tumor cells release many damage-associated molecules (DAMs), including double-stranded DNA (dsDNA), dsRNA, and single-stranded RNA (ssRNA), under anaerobic and esurient conditions. These DAMs stimulate innate and adaptive immune responses by interacting with their pattern recognition receptors (PRRs) 55. In these processes, dsDNA sensors, such as the cGAS/STING axis, and RNA susceptors, including several Toll-like receptors (TLRs), RIG-1 and MDA5, contribute to activating the production of type I interferon, which strengthens the antitumor immune response or induces PD-L1-mediated immunotolerance 56, 57. A recent study showed that ablation of KLF5 reduced the mRNA levels of *STING* and *MDA5* 58. KLF5 was hypothesized to be responsible for sustaining high PD-L1 expression by increasing *STING* and *MDA5* transcription, which resisted the immune killing effect. Ultimately, KLF5 modulated the secretion of various inflammatory chemokine factors. KLF5 silencing lessened the mRNA expression and release of interleukin 6 (IL6) and IL8 59. Likewise, an acetylation-mimicking mutant of KLF5 resulted in a marked increase in cancer-promoting IL18, IL6 and IL11 60, and acetylated KLF5 functioned as a tumor suppressor 48. Mechanistically, unacetylated KLF5 inhibits the activity of STAT1 and STAT3, two main transcription factors of inflammatory chemokine factors 61, 62. Our transcriptomic analysis suggested that CXCL5 was elevated in the KLF5^low^ group. Likewise, p300-acetylated KLF5 was reported to increase *CXCL5* transcription 25. The potential mechanism may be that acetylated KLF5 is prone to ubiquitination and degradation 63. Therefore, KLF5 contributes to the formation of a protumorigenic microenvironment by facilitating the release of inflammatory factors.

The COX2/PGE2 pathway is a key determinant of the inflammatory response. However, the influence of KLF5 on this pathway remains unclear. Initially, KLF5 deletion reduces *COX2* mRNA expression, further inhibiting the release of PGE2 and PGF2a 59. Furthermore, KLF5 binds to the *COX2* gene promoter to increase *COX2* expression at the transcriptional level 39. In the present study, COX2-associated lncRNAs (*Ptgs2os2* and *Ptgs2os*) were positively correlated with Klf5 expression. These lncRNAs activated the transcription of *COX2* (encoding the* Ptgs2* gene) in an RNA-enhancing manner 64. LncRNAs may mediate KLF5-activated COX2 expression. As a key enzyme, mPGES1 directly converts PGG2 or PGH2 to PGE2, and it is highly expressed in TNBC 14. Our previous study demonstrated that *mPGES1* is a direct target gene of KLF5, and inhibition of KLF5/mPGES1 signaling decreased the conversion of PGE2 from PGH2^14^. Hence, KLF5 likely contributes to PGE2 production twofold. Although Ptgs2 inductions in cDC1s contributes to CD8^+^T cell expansion, they have just examined the impact of Ptgs2 on the priming stage for anti-tumor immunity, rather than in the TME, which involves many distinct processes 65. In TMEs, the COX2/PGE2 axis in tumor cells or stromal cells are both equipped with immunosuppression 32, 33, 66. With growing interest in the interactions between stromal cells and immune cells 67, there has been reported that COX2^+^ lung adventitial fibroblasts (AdvFs) drive myeloid cell dysfunction or immunosuppression. Furthermore, Tumor-driven IL-1b reinforces myeloid cell reprogramming by COX2^+^ lung AdvFs 68. Mechanically, PGE2 induces CXCL12 expression to recruit MDSCs by interacting with its receptor CXCR4 69. Additionally, PGE2 blocks the differentiation of monocytes to dendritic cells (DCs) but redirects monocytes developing to MDSCs 70. Furthermore, PGE2 promotes PD-L1 expression on tumor-associated macrophages and MDSCs 71. Overall, PGE2 promotes the recruitment and activation of immunosuppressive cells to destroy antitumor immunity. Similarly, PGE2 directly impairs antitumor effector cells, including NK cells and T cells. Deletion of PGE2 receptors on NK cells enhances the cytotoxic activity of NK cells and further activates the T-cell-mediated adaptive antitumor immune response 33. During the process, NK cells secrete CXCL1 and CCL5, which recruit conventional type 1 dendritic cells (cDC1) and CD8^+^ effector T cells, respectively. Furthermore, cDC1 stimulates the proliferation and functionality of CD8^+^ effector T cells by releasing IL12. Consistently, genetic silencing of KLF5 in tumors resulted in a marked increase in CXCL1, CCL5 and their receptors and enhanced IL12 production. Consequently, the KLF5/COX2 pathway may destroy NK cells and T-cell-modulates antitumor immunity by producing PGE2.

Targeting the KLF5/COX2/PGE2 axis may be an effective therapeutic strategy in diverse cancers, including BLBC. Mifepristone is an effective inhibitor of KLF5^24^. Similarly, mifepristone led to immunogenic cell death of tumor cells, subsequently increasing the infiltration of MHC-II^+^ DCs, natural killer cells and CD8^+^ central memory T cells to sensitize the tumors to anti-PD1 blockers 72. Given this evidence, anti-inflammatory drugs targeting COX2 or mPGES1 succeeded in improving immune escape and synergizing with the efficacy of ICBs 32, 33, 73, 74. Mechanistically, inhibition of COX2 or mPGES1 decreased the infiltration of MDSCs but increased the number and functions of cytotoxic cells such as NK cells and CD8^+^ T cells. Because COX2 inhibitors have cardiac side effects, blocking PGE2 receptors may be a promising method. Several inhibitors targeting EP2 or EP4 have been found to potentiate anti-PD1 efficacy and shift the “cold” to the “hot” tumor microenvironment 34, 66, 75.

In summary, our results indicate the potential of the KLF5/COX2/PGE2 axis as a therapeutic target to improve the efficacy of ICBs in BCLC and other cancers.

## Conclusions

In conclusion, the present findings decipher the effect of KLF5-induced PGE2 generation modulation on cancer immune escape, highlighting an immunostimulatory role of KLF5 inhibitors for cancer therapy. Furthermore, KLF5 blockers in combination with ICBs may provide a novel therapy in cancer immunotherapy.

## Abbreviations

ICBs: Immune checkpoint blockers
TME: tumor microenvironment
KLF5: Krüppel-like Factor 5
ChIP: chromatin immunoprecipitation
PGE2: prostaglandin E2
KC-IS: KLF5/COX2-associated immune score
TNBC: triple-negative breast cancer
TIME: tumor-immune microenvironment
