# Supplementary material for: KLF5 inhibition potentiates anti-PD1 efficacy by enhancing CD8+ T-cell-dependent antitumor immunity
Source: Theranostics. 2023 Feb 21;13(4):1381–400. doi: 10.7150/thno.82182 (PMC10008740; doi:10.7150/thno.82182)
Supplement: Supplementary file 1 — Supplementary figures and tables. [file thnov13p1381s1.pdf]

mKLF5 cDNA	ATGCCCACGCGGGTGCTGACCATGAGCGCCCGCCTGGGACCACTGCCCCAGCCGCCGGCCGC<br>GCAGGACGAGCCCGTGTTCGCGCAGCTCAAGCCGGTGCTGGGCGCTGCGAACCCGGCCCGCG<br>ACGCGGCGCTCTTCTCCGGAGACGATCTGAAACACGCGCACCACCACCCGCCTGCGCCGCCG<br>CCAGCCGCTGGCCCGCGACTGCCCTCGGAGGAGCTGGTCCAGACAAGATGTGAAATGGAGAA<br>GTATCTGACCCCTCAGCTCCCTCCAGTTCCGATAATTTCAGAGCATAAAAAGTATAGACGAG<br>ACAGTGCCTCAGTGGTAGACCAGTTCTTCACTGACACTGAAGGCATACCTTACAGCATCAAC<br>ATGAACGTCTTCCTCCCTGACATCACTCACCTGAGAACTGGCCTCTACAAATCCCAGAGACC<br>ATGCGTAACACAGATCAAGACAGAACCTGTTACCATTTTCAGCCACCAGAGCGAGTCGACGG<br>CCCCTCCTCCTCCTCCGGCCCCCACCCAGGCTCTCCCCGAGTTCACTAGTATCTTCAGCTCC<br>CACCAGACCACAGCGCCACCACAGGAGGTGAACAATATCTTCATCAAACAAGAACTTCCTAT<br>ACCAGATCTTCATCTCTCTGTCCCTTCCCAGCAGGGCCACCTGTACCAGCTGTTGAATACAC<br>CGGATCTAGACATGCCCAGTTCGACAAACCAGACGGCAGTAATGGACACCCTTAATGTTTCT<br>ATGGCAGGCCTTAACCCACACCCCTCTGCTGTTCCACAGACGTCAATGAAACAGTTCCAGGG<br>CATGCCCCCTTGCACGTACACCATGCCAAGTCAGTTTCTTCCACAGCAGGCCACCTACTTTC<br>CCCCGTCACCACCAAGCTCAGAGCCTGGAAGTCCCGATAGACAAGCTGAGATGCTGCAGAAT<br>CTCACCCCACCTCCGTCCTATGCCGCTACAATTGCTTCCAAACTGGCGATTCACAACCCAAA<br>TTTACCTGCCACTCTGCCAGTTAATTCGCCAACTCTCCCACCTGTCAGATACAACAGAAGGA<br>GTAACCCGGATCTGGAGAAGCGACGTATCCACTTCTGCGATTATAATGGTTGCACAAAAGTT<br>TATACAAAGTCGTCTCACTTAAAAGCTCACCTGAGGACTCATACGGGCGAGAAGCCCTACAA<br>GTGCACCTGGGAGGGCTGCGACTGGAGGTTTGCCCGGTCGGATGAGCTGACCCGCCACTACA<br>GGAAGCACACGGGCGCCAAGCCGTTCCAGTGCATGGTGTGCCAACGCAGCTTCTCCCGCTCC<br>GACCACCTCGCGCTGCACATGAAGCGCCACCAGAAC

Table S1. The sequences of plasmids and lentiviruses.

Table S2. The primers sequences of mRNA.

Name	Forward sequences	Reverse sequences
**mPTGS2**	CATCCCCTTCCTGCGAAGTT	GGCCCTGGTGTAGTAGGAGA
**mGAPDH**	AGGTCGGTGTGAACGGATTTG	GGGGTCGTTGATGGCAACA

Table S3 Genes related to KC-IS

Cancer promoting inflammatory genes	Cancer inhibitory inflammatory genes
IL17RE	CD28
RAC3	CD4
CNTF	PLA2G2D
PLA2G4A	CRTAM
PTGS2	CD3E
ALOX12	CARD11
	RASAL3
	ZAP70
	EBI3
	TBX21
	IKZF1
	EOMES
	LCK
	CD3G
	CD8A
	THEMIS
	CD3D
	CD27
	ITK
	LEF1
	FLT3
	IRF4
	PTGER4
	SLAMF6
	KLRK1
	CD96
	CD226
	CD2
	IL1RL1
	CD247
	IL16
	NLRC3
	CCL17
	CCL22
	CXCL13
	XCR1
	CCR7

	CXCR3
	CXCR6
	CX3CR1
	LIFR
	TNFSF8
	CCRL2
	STAP1

Table S4 Patient characteristic

Clinicopathological parameters		NO. of cases N=67
Survival time (months)		38.1±12.7
Age at diagnosis, y		
	＜60	23
	⩾60	44
Gender		
	male	34
	female	33
Size (largest diameter,cm)		
	⩽5	26
	＞5	23
	unknown	18
Location		
	Ascending/Transverse	39
	Descending/Sigmoid	28
Lymph node metastasis		
	negative	45
	positive	22
AJCC clinical stage		
	0	1
	1	6
	2	30
	3	21
	4	3
	unknown	6
Recurrence		
	no	56
	yes	11
KLF5		
	negative	10
	positive	57
CD8		
	negative	43
	positive	24

Figure S1. Related to Figure 1.

(**A**) Schematic of the experimental setup. (**B**) The expression of Klf5 was screened in multiple tumor cells. (**C-E**). The Klf5 level in PyMT-induced tumor cells was verified by Western blotting (**C**); representative images of tumors are displayed in (**D**); tumor sizes are quantified in (**E**). (**F-H**) Klf5 expression was silenced by siRNA lentivirus in EMT6 cells. Furthermore, the levels of Klf5 and its target gene cyclin D1 were verified by Western blotting (**F**). Representative images of tumors are displayed in (**G**). Tumor sizes are quantified in (**H**). (**I-K**) 67NR cells were transfected with lentivirus carrying Klf5. The levels of Klf5 and its target gene CyclinD1 were then verified by Western blotting (**F**); representative images of tumors are displayed in (**G**); tumor sizes are quantified in (**H**). Plot of tumor size (means ± SD) (*p < 0.05 or ns, not statistically significant vs. control; Student's t test).

Figure S2. Activation of Klf5 is associated with an immunosuppressive microenvironment.

BALB/c mice were inoculated with control or Klf5-overexpressing (OV) 67NR cells. These tumors were collected for RNA transcriptional sequencing when they grew to approximately 1 cm. (**A**) Volcano plot of differentially expressed genes in Klf5-overexpressing versus control 67NR tumors. Significant gene expression with changes > 1.5-fold and P <0.05 was considered. (**B**) Gene Ontology (GO) analysis was performed on transcriptional profiles for Klf5 OV vs. control groups. (**C**) Heatmap of the differential expression of immune-related genes in Klf5-overexpressing versus control 67NR tumors. (**D**) The Xcell method was performed to define the immune cell populations in Klf5-overexpressing versus control 67NR tumors. Significantly activated molecules or cell populations are highlighted in red. *p < 0.05.

Figure S3. Related to Figure 3.

(**A**) Western blot analysis of KLF5, CyclinD1 and COX2 in both KLF5-deficient and control HCC1806 cells. (**B**) HEK293T cells were cotransfected with COX2 (−1100/+100)-luc or COX2mut (−1100/+100)- luc plus PCDH-KLF5 or control vector PCDH and the internal control plasmid pRL-TK. (**C-D**) HCC1806 cells were subjected to the ChIP assay using the anti-KLF5 antibody or control goat IgG. PCR was performed to amplify regions surrounding the putative KLF5 binding region and a nonspecific KLF5 binding region. (**E**) Relative Ptges mRNA expression in control and Klf5-deficient EMT6 cells. (**F**) Relative Ptges mRNA expression in control and Klf5-overexpressing 67NR cells. (G) Western blot analysis of Klf5 and Ptges in both Klf5-deficient or control EMT6 and Klf5-overexpressing or control 67NR cells.

Figure S4. Blocking the Klf5/Cox2 axis synergizes with the efficacy of anti-PD1 therapy in a colon cancer model.

(**A**) Schematic of the experimental setup. The mice received the mentioned treatments when tumors were approximately 20 mm^2 in mean area. Growth curves (mean ± SEM), tumor size distributions at Day 23, individual tumor growth curves and survival curves are shown. $n \geq 4$ for mice in each group. (*p < 0.05; **, p <0.01; ***, p <0.001; ****, p <0.0001 or not statistically significant vs. control; two-way ANOVA).

Figure S5. Related to Figure 6.

(**A-B**) Bubble heatmap of the expression of cell type-specific genes within the interface subclusters. Dot size indicates the fraction of expressing cells, colored based on normalized expression levels. (**C, E**) Different spatial distributions of KRT14, KRT17 KRT5, KLF5, CD4 and CD8 were overlaid onto tissue spots from our previous study or public dataset. (**D**) Immunohistochemical images of KLF5, COX2 and CD8 in the ST sample from our previous study. (**F**) Box plots show the enrichment scores of the basal signature, CD4 signature and CD8 signature in the $KLF5^{high}$ and $KLF5^{low}$ groups from the public dataset.

Figure S6. Related to Figure 7.

(**A**) Expression of KLF5 and PTGS2 in TNBC subtypes as defined in a previous study. (**B**) Enrichment of Gene Ontology (GO) analysis to estimate the biological processes in transcriptional profiles for $KLF5^{low}$ vs. $KLF5^{high}$ groups. (**C-D**) The levels of Klf5 and Cd8 were quantified by ImageJ after staining with specific antibodies in paraffin-embedded samples obtained from 67 patients with colon cancer. Representative images of Klf5 and Cd8 (**B**). The scale bar indicates 50 μm. (**C**) Kaplan–Meier survival analysis of patients in the double-positive group versus the double-negative group.

Figure S1

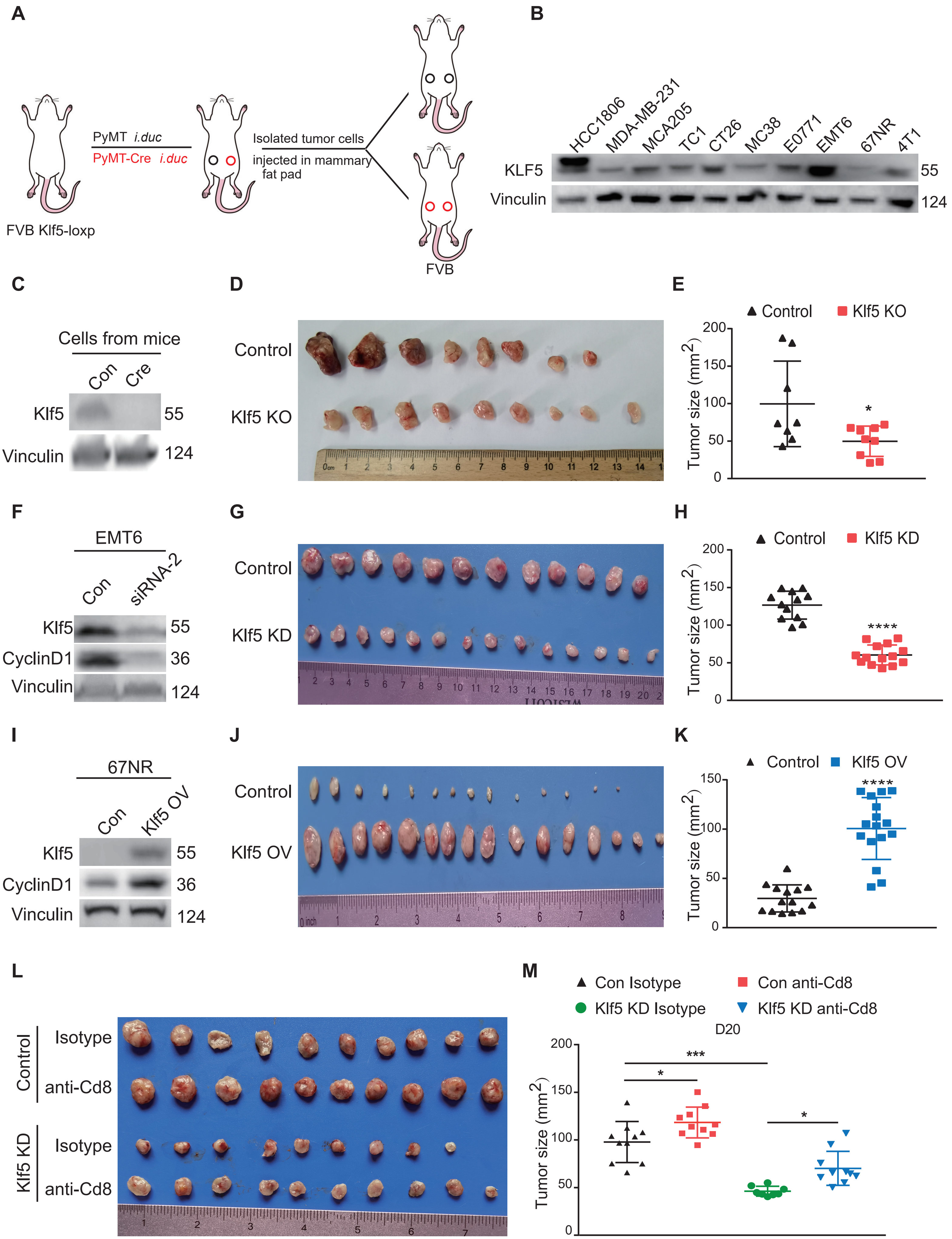
A
PyMT i.duc
PyMT-Cre i.duc
Isolated tumor cells
injected in mammary fat pad
FVB Klf5-loxp
FVB
B
HCC1806
MDA-MB-231
MCA205
TC1
CT26
MC38
E0771
EMT6
67NR
4T1
KLF5
Vinculin
55
124
C
Cells from mice
Con
Cre
Klf5
Vinculin
55
124
D
Control
Klf5 KO
E
Control
Klf5 KO
Tumor size (mm2)
*
F
EMT6
Con
siRNA-2
Klf5
CyclinD1
Vinculin
55
36
124
G
Control
Klf5 KD
H
Control
Klf5 KD
Tumor size (mm2)
****
I
67NR
Con
Klf5 OV
Klf5
CyclinD1
Vinculin
55
36
124
J
Control
Klf5 OV
K
Control
Klf5 OV
Tumor size (mm2)
****
L
Control
Isotype
anti-Cd8
Klf5 KD
Isotype
anti-Cd8
M
Con Isotype
Con anti-Cd8
Klf5 KD Isotype
Klf5 KD anti-Cd8
D20
Tumor size (mm2)
***
*
*

Figure S2

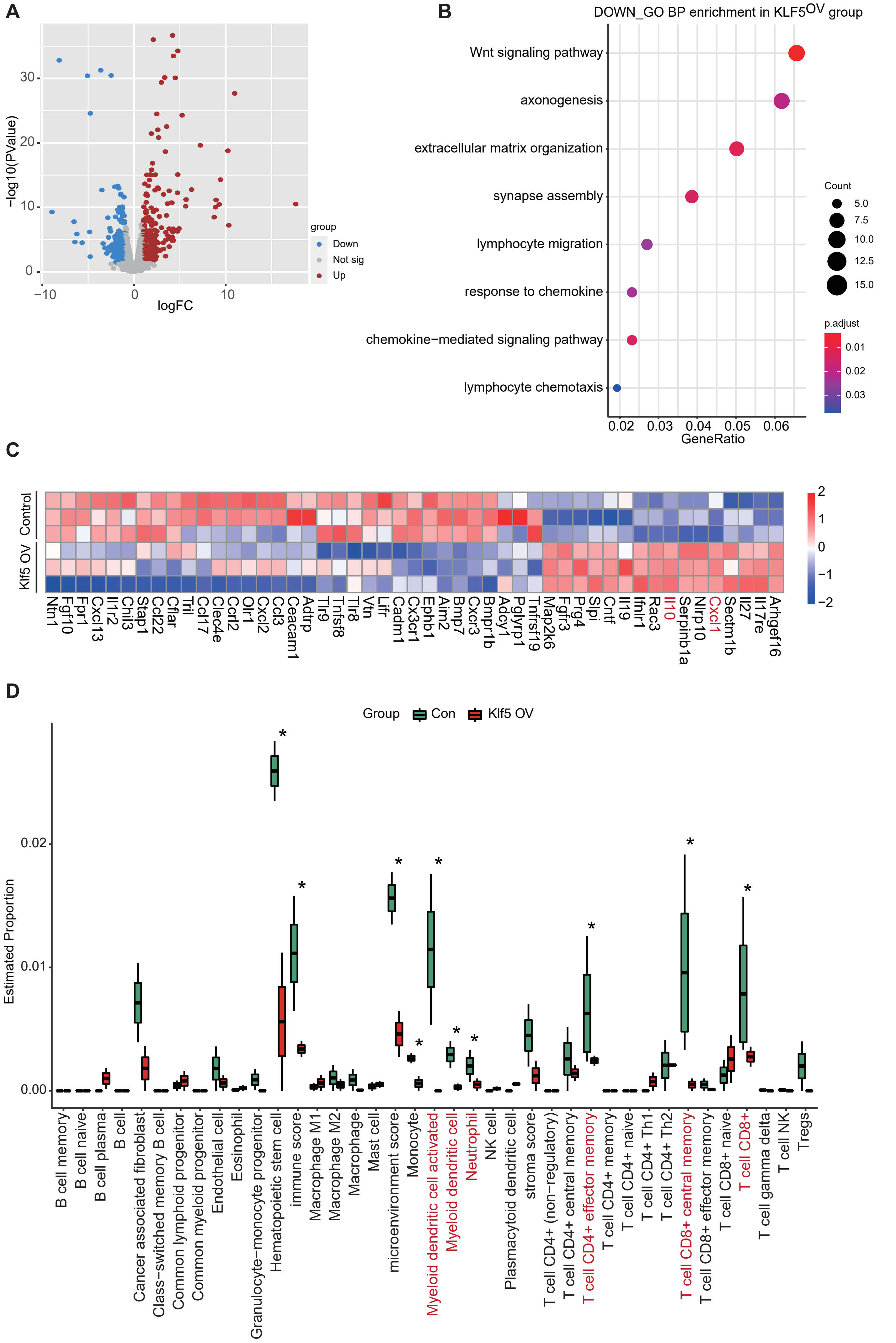

A
−log10(PValue)
logFC
group
Down
Not sig
Up
B
DOWN_GO BP enrichment in KLF5OV group
Wnt signaling pathway
axonogenesis
extracellular matrix organization
synapse assembly
lymphocyte migration
response to chemokine
chemokine−mediated signaling pathway
lymphocyte chemotaxis
GeneRatio
Count
p.adjust
C
Control
Klf5 OV
Ntn1 Fgf10 Fpr1 Cxcl13 Il1r2 Chil3 Stap1 Ccl22 Cflar Tril Ccl17 Clec4e Ccrl2 Olr1 Cxcl2 Ccl3 Ceacam1 Adtrp Tlr9 Tnfsf8 Tlr8 Vtn Lifr Cadm1 Cx3cr1 Ephb1 Aim2 Bmp7 Cxcr3 Bmpr1b Adcy1 Pglyrp1 Tnfrsf19 Map2k6 Fgfr3 Prg4 Slpi Cntf Il19 Ifnlr1 Rac3 Il10 Serpinb1a Nlrp10 Cxcl1 Sectm1b Il27 Il17re Arhgef16
D
Group
Con
Klf5 OV
Estimated Proportion
B cell memory
B cell naive
B cell plasma
B cell
Cancer associated fibroblast
Class−switched memory B cell
Common lymphoid progenitor
Common myeloid progenitor
Endothelial cell
Eosinophil
Granulocyte−monocyte progenitor
Hematopoietic stem cell
immune score
Macrophage M1
Macrophage M2
Macrophage
Mast cell
microenvironment score
Monocyte
Myeloid dendritic cell activated
Myeloid dendritic cell
Neutrophil
NK cell
Plasmacytoid dendritic cell
stroma score
T cell CD4+ (non−regulatory)
T cell CD4+ central memory
T cell CD4+ effector memory
T cell CD4+ memory
T cell CD4+ naive
T cell CD4+ Th1
T cell CD4+ Th2
T cell CD8+ central memory
T cell CD8+ effector memory
T cell CD8+ naive
T cell CD8+
T cell gamma delta
T cell NK
Tregs

Figure S3

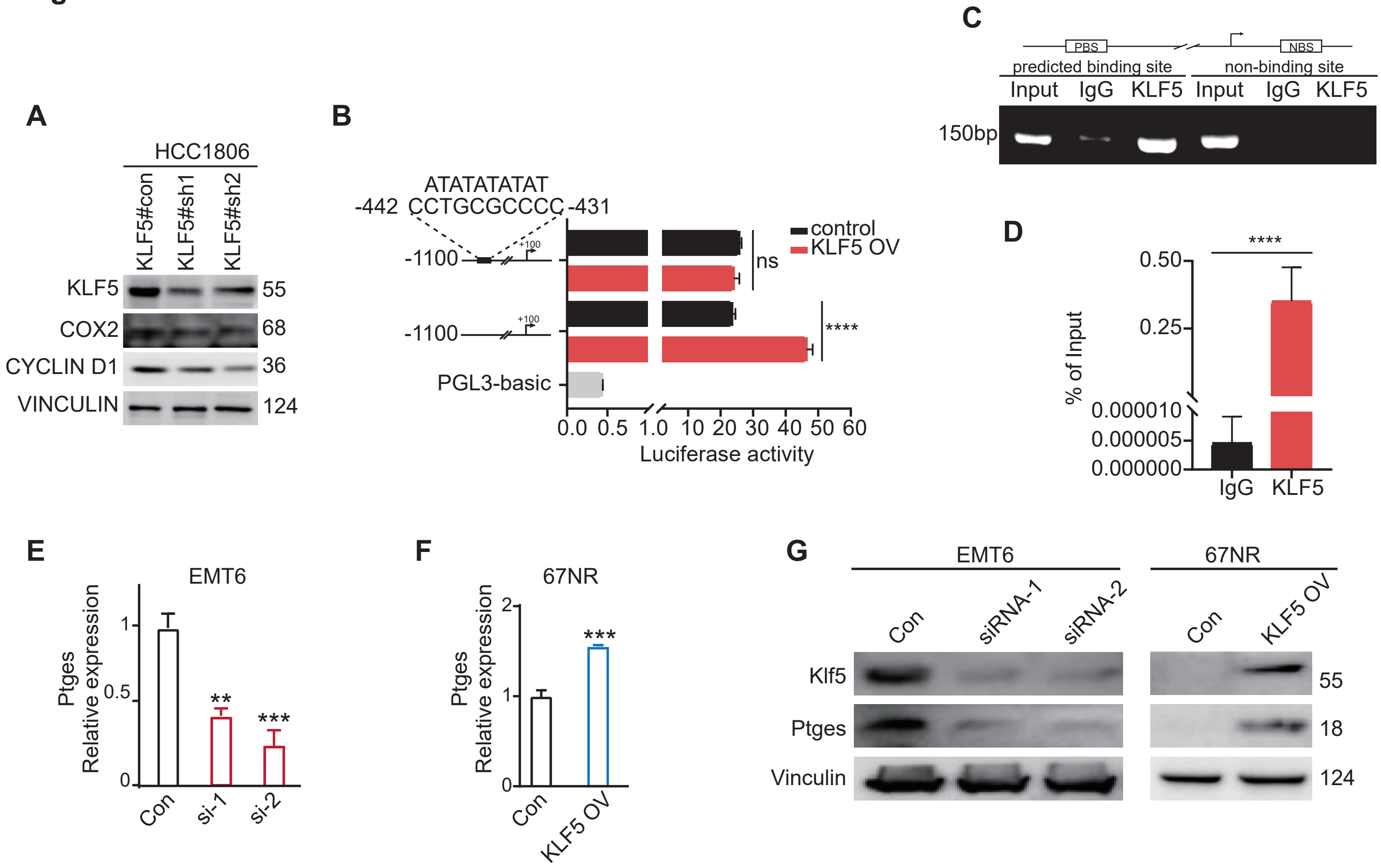
A
HCC1806
KLF5#con
KLF5#sh1
KLF5#sh2
KLF5
COX2
CYCLIN D1
VINCULIN
55
68
36
124
B
ATATATATAT
-442 CCTGCGCCCC -431
-1100
+100
-1100
PGL3-basic
control
KLF5 OV
ns
****
0.0 0.5 1.0 10 20 30 40 50 60
Luciferase activity
C
PBS
NBS
predicted binding site
non-binding site
Input IgG KLF5 Input IgG KLF5
150bp
D
****
0.50
0.25
0.000010
0.000005
0.000000
% of Input
IgG
KLF5
E
EMT6
Ptges Relative expression
1
0.5
0
**
***
Con
si-1
si-2
F
67NR
Ptges Relative expression
2
1
0
***
Con
KLF5 OV
G
EMT6
67NR
Con
siRNA-1
siRNA-2
Con
KLF5 OV
Klf5
Ptges
Vinculin
55
18
124

Figure S4

A

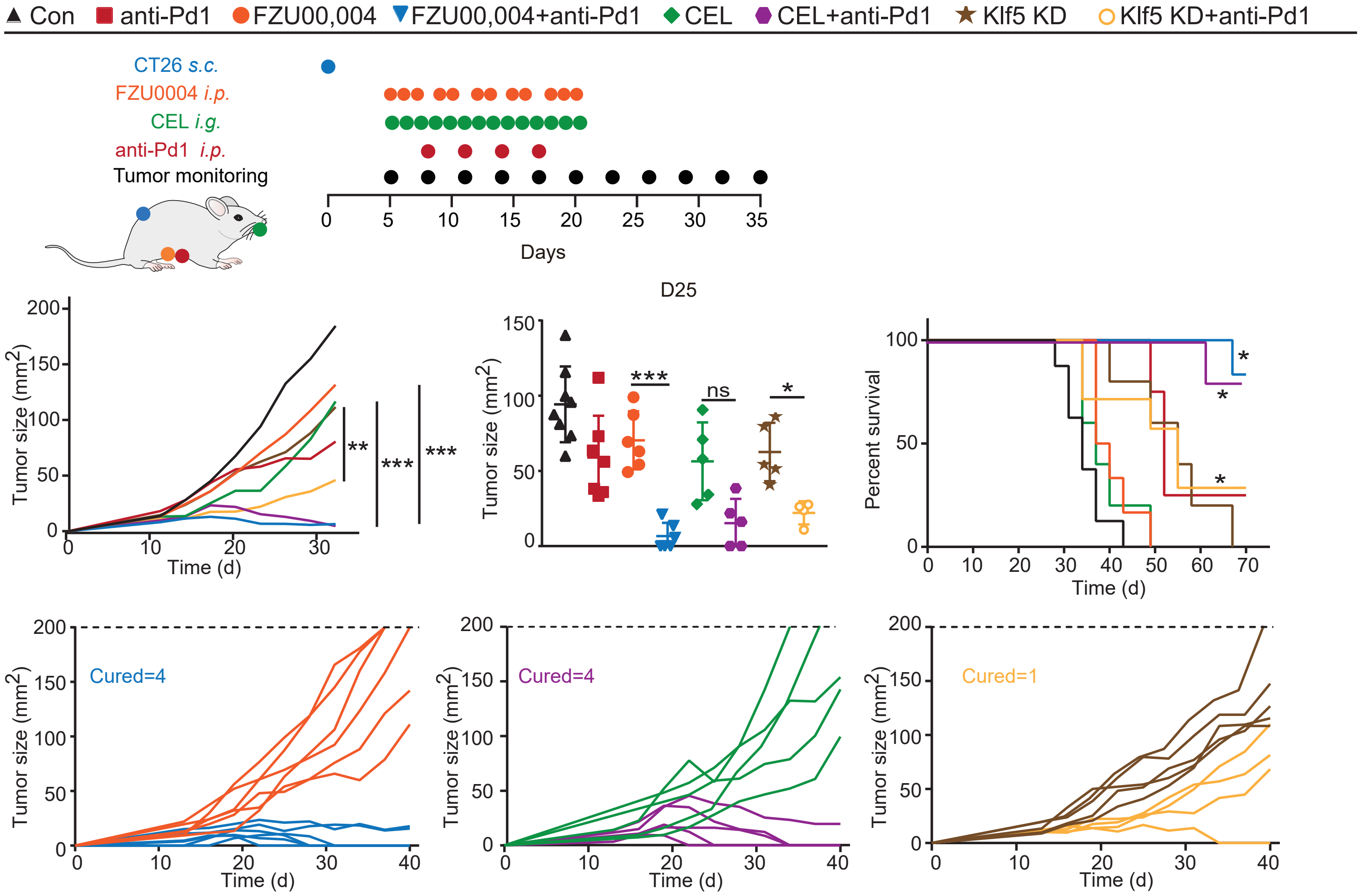

Con
anti-Pd1
FZU00,004
FZU00,004+anti-Pd1
CEL
CEL+anti-Pd1
Klf5 KD
Klf5 KD+anti-Pd1
CT26 *s.c.*
FZU0004 *i.p.*
CEL *i.g.*
anti-Pd1 *i.p.*
Tumor monitoring
Days
D25
Tumor size (mm2)
Time (d)
Percent survival
**
***
ns
*
Cured=4
Cured=4
Cured=1

Figure S5

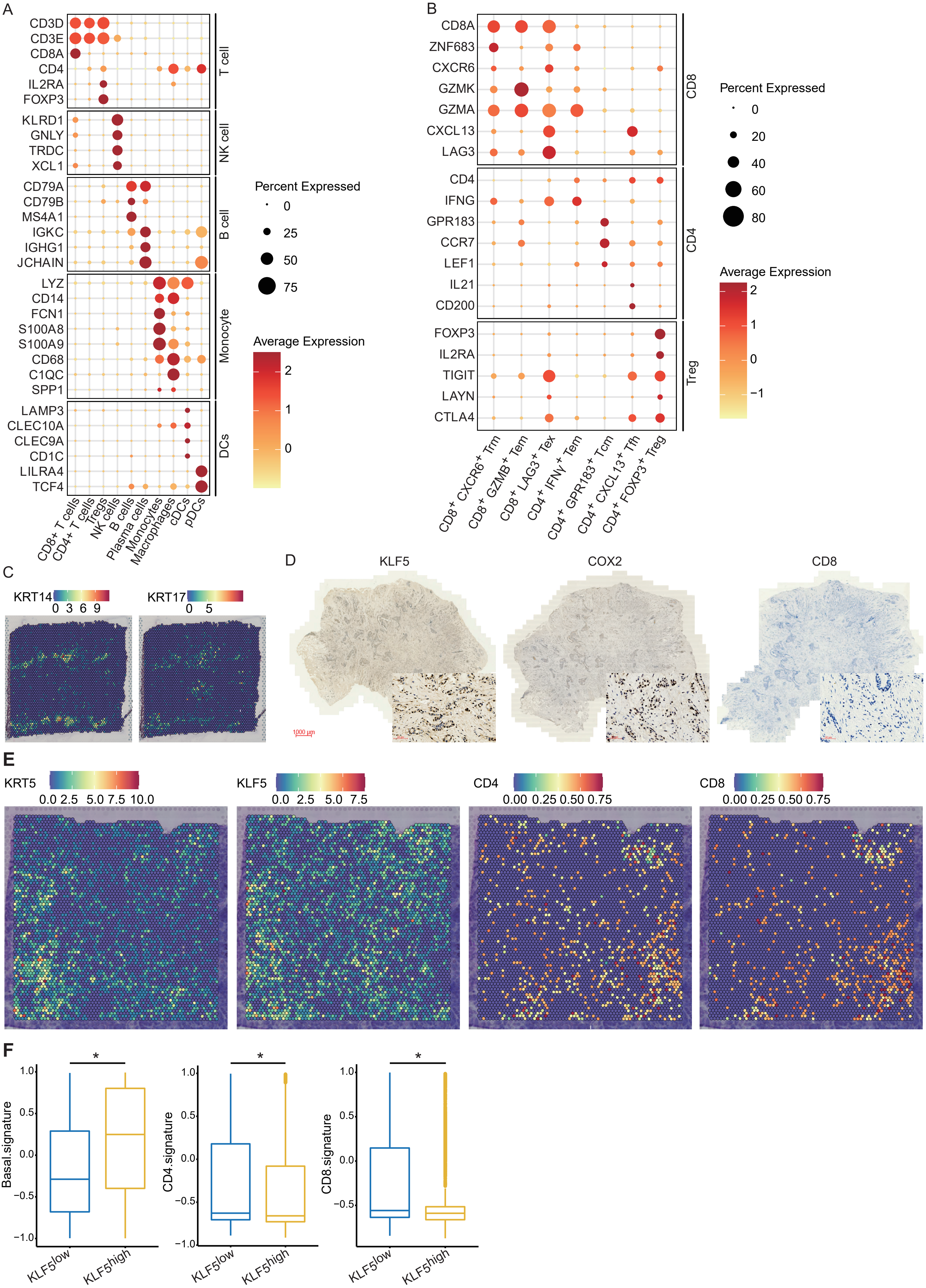
A
CD3D
CD3E
CD8A
CD4
IL2RA
FOXP3
KLRD1
GNLY
TRDC
XCL1
CD79A
CD79B
MS4A1
IGKC
IGHG1
JCHAIN
LYZ
CD14
FCN1
S100A8
S100A9
CD68
C1QC
SPP1
LAMP3
CLEC10A
CLEC9A
CD1C
LILRA4
TCF4
T cell
NK cell
B cell
Monocyte
DCs
CD8+ T cells
CD4+ T cells
Tregs
NK cells
B cells
Plasma cells
Monocytes
Macrophages
cDCs
pDCs
Percent Expressed
0
25
50
75
Average Expression
2
1
0
B
CD8A
ZNF683
CXCR6
GZMK
GZMA
CXCL13
LAG3
CD4
IFNG
GPR183
CCR7
LEF1
IL21
CD200
FOXP3
IL2RA
TIGIT
LAYN
CTLA4
CD8
CD4
Treg
CD8+ CXCR6+ Trm
CD8+ GZMB+ Tem
CD8+ LAG3+ Tex
CD4+ IFNγ+ Tem
CD4+ GPR183+ Tcm
CD4+ CXCL13+ Tfh
CD4+ FOXP3+ Treg
Percent Expressed
0
20
40
60
80
Average Expression
2
1
0
−1
C
KRT14
0 3 6 9
KRT17
0 5
D
KLF5
COX2
CD8
1000 μm
E
KRT5
0.0 2.5 5.0 7.5 10.0
KLF5
0.0 2.5 5.0 7.5
CD4
0.00 0.25 0.50 0.75
CD8
0.00 0.25 0.50 0.75
F
Basal.signature
CD4.signature
CD8.signature
1.0
0.5
0.0
−0.5
−1.0
*
KLF5low
KLF5high

Figure S6

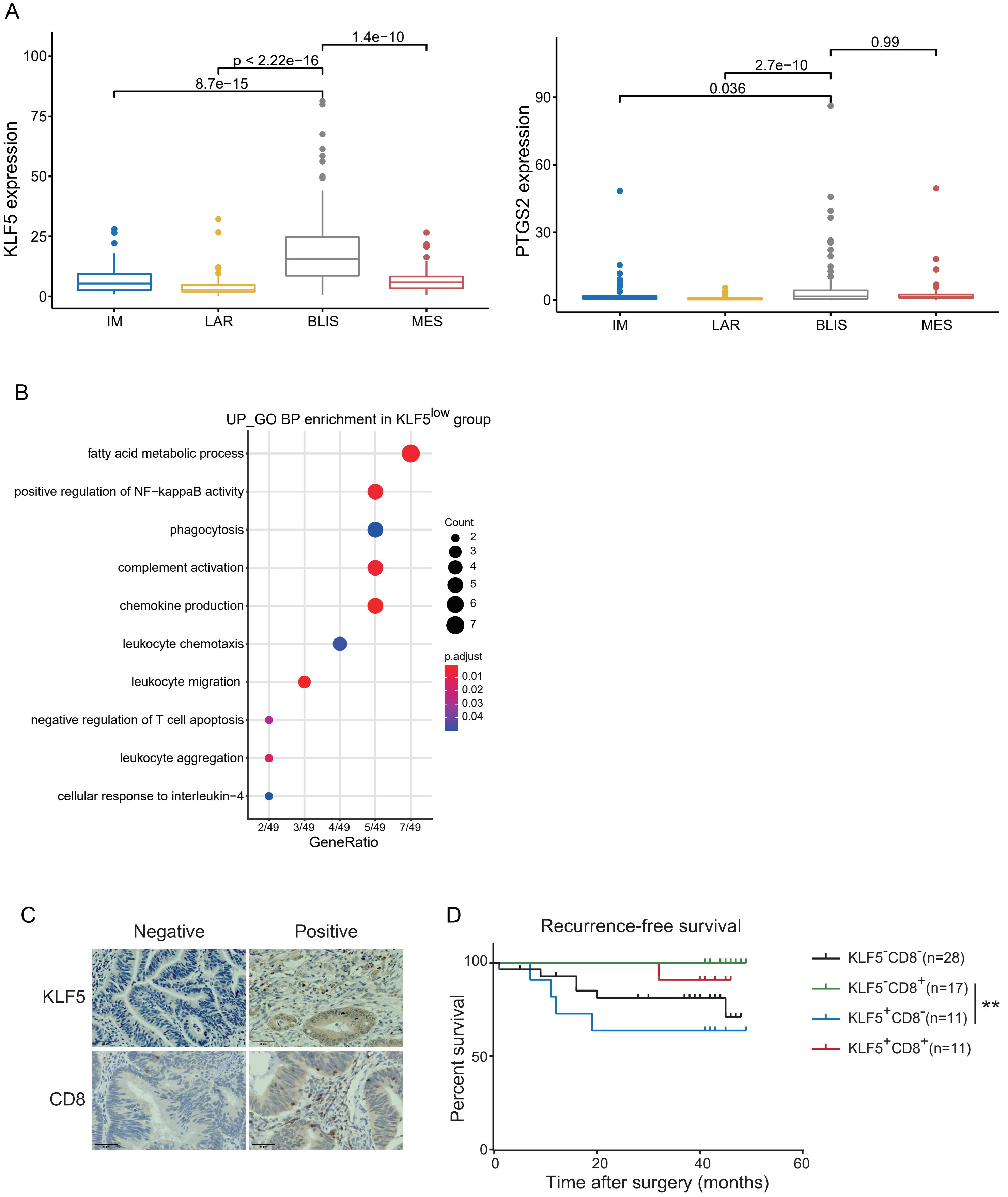

A
KLF5 expression
IM
LAR
BLIS
MES
8.7e−15
p < 2.22e−16
1.4e−10
PTGS2 expression
0.036
2.7e−10
0.99
B
UP_GO BP enrichment in KLF5low group
fatty acid metabolic process
positive regulation of NF−kappaB activity
phagocytosis
complement activation
chemokine production
leukocyte chemotaxis
leukocyte migration
negative regulation of T cell apoptosis
leukocyte aggregation
cellular response to interleukin−4
2/49
3/49
4/49
5/49
7/49
GeneRatio
Count
p.adjust
C
Negative
Positive
KLF5
CD8
D
Recurrence-free survival
Percent survival
Time after surgery (months)
KLF5-CD8-(n=28)
KLF5-CD8+(n=17)
KLF5+CD8-(n=11)
KLF5+CD8+(n=11)
**
